# Turbulence in a small boreal lake: Consequences for air–water gas exchange

**DOI:** 10.1002/lno.11645

**Published:** 2020-11-24

**Authors:** Sally MacIntyre, David Bastviken, Lars Arneborg, Adam T. Crowe, Jan Karlsson, Andreas Andersson, Magnus Gålfalk, Anna Rutgersson, Eva Podgrajsek, John M. Melack

**Affiliations:** ^1^ Department of Ecology, Evolution and Marine Biology University of California Santa Barbara CA USA; ^2^ Marine Science Institute, University of California Santa Barbara CA USA; ^3^ Earth Research Institute, University of California Santa Barbara CA USA; ^4^ Department of Thematic Studies—Environmental Change Linköping University Linköping Sweden; ^5^ Swedish Meteorological and Hydrological Institute Vastra Frolunda Sweden; ^6^ Department of Ecology and Environmental Science Umeå University Umeå Sweden; ^7^ Uppsala University Uppsala Sweden; ^8^ Department of Ecotechnology and Sustainable Building Engineering Mid Sweden University Östersund Sweden

## Abstract

The hydrodynamics within small boreal lakes have rarely been studied, yet knowing whether turbulence at the air–water interface and in the water column scales with metrics developed elsewhere is essential for computing metabolism and fluxes of climate‐forcing trace gases. We instrumented a humic, 4.7 ha, boreal lake with two meteorological stations, three thermistor arrays, an infrared (IR) camera to quantify surface divergence, obtained turbulence as dissipation rate of turbulent kinetic energy (*ε*) using an acoustic Doppler velocimeter and a temperature‐gradient microstructure profiler, and conducted chamber measurements for short periods to obtain fluxes and gas transfer velocities (*k*). Near‐surface *ε* varied from 10^−8^ to 10^−6^ m^2^ s^−3^ for the 0–4 m s^−1^ winds and followed predictions from Monin–Obukhov similarity theory. The coefficient of eddy diffusivity in the mixed layer was up to 10^−3^ m^2^ s^−1^ on the windiest afternoons, an order of magnitude less other afternoons, and near molecular at deeper depths. The upper thermocline upwelled when Lake numbers (*L*_*N*_) dropped below four facilitating vertical and horizontal exchange. *k* computed from a surface renewal model using *ε* agreed with values from chambers and surface divergence and increased linearly with wind speed. Diurnal thermoclines formed on sunny days when winds were < 3 m s^−1^, a condition that can lead to elevated near‐surface *ε* and *k*. Results extend scaling approaches developed in the laboratory and for larger water bodies, illustrate turbulence and *k* are greater than expected in small wind‐sheltered lakes, and provide new equations to quantify fluxes.

Small‐ and moderate‐sized lakes are abundant and widespread in boreal regions throughout Europe, North America, and Asia (Verpoorter et al. 2014). Many of these lakes are supersaturated with CO_2_ and outgassing from the lakes represents a significant portion of imported organic and inorganic carbon (Sobek et al. 2003; Vachon et al. 2016; Hessen et al. 2017). The magnitude of carbon fluxes depends on whether the lakes are autotrophic or net heterotrophic and on ongoing changes in land use and climate. Carbon fluxes also depend on hydrodynamic processes, which moderate concentration gradients of dissolved gases including those that transport dissolved gases to the air–water interface and those which mediate transfer across the air–water interface. Within the water column, vertical fluxes are caused by turbulence, often from enhanced shear across the thermocline or by entrainment during cooling events. At the air–water interface, fluxes are mediated by small‐scale upwelling or divergence events which can be quantified based on turbulence or by resolving the near‐surface flows indicative of these processes (Lamont and Scott 1970; MacIntyre et al. 1995; Wang et al. 2015). Turbulent processes in the water column are quantified with the coefficient of eddy diffusivity (*K*_*z*_), those at the air–water interface by gas transfer velocities (*k*), and horizontal spreading by a dispersion coefficient (*K*_H_). Understanding controls on the spatial and temporal variability of turbulence and horizontal exchange is essential for calculating fluxes and lake metabolism.

Several attributes of small boreal lakes may modify their mixing dynamics relative to larger or clearer lakes. High concentrations of chromophoric dissolved organic matter (CDOM) are typical for small boreal lakes as is sheltering from wind. These conditions are conducive to shallow upper mixed layers, formation of near‐surface stratification and diurnal mixed layers, and strongly stratified thermoclines (Xenopoulos and Schlinder 2001; Houser 2006). Although these conditions suggest turbulence may be reduced within the water column, near‐surface turbulence can be augmented under conditions of heating with light winds (Wyngaard and Coté 1971; Tedford et al. 2014). The high concentrations of CDOM and the resultant increased near‐surface heating will likely lead to the temperature of surface water being higher than that of air and an unstable atmosphere above the lake. These conditions can lead to greater momentum transfer and appreciable turbulence even under light to moderate winds (MacIntyre et al. 2018). Shear and mixing may be enhanced at the base of the mixed layer or top of the thermocline when diurnal mixed layers are present (Imberger 1985), when the upper mixed layer is shallow (Antenucci and Imberger 2001; Boegman et al. 2003), or when mixed layer depth is a small fraction of mean depth (Imberger and Patterson 1990; Horn et al. 2001). Alternatively, reduced mixing may enable a longer duration of horizontal flows and concomitant inshore‐offshore exchange in the mixed layer and thermocline due to wind‐driven circulation and seiching (Mortimer 1952, 1961). Mixing driven by heat loss may predominate over that from wind shear in transporting dissolved gases to the air–water interface (Crill et al. 1988; Aberg et al. 2010; Liu et al. 2016). Thus, despite diurnal mixed layers and strongly stratified thermoclines, several attributes of boreal lakes may facilitate near‐surface turbulence and within lake exchanges.

Evaluation of scaling approaches based on general principles can indicate whether equations to estimate near‐surface and within‐lake turbulence hold independently of lake size and mixed layer depth. Monin–Obukov similarity theory (MOST) predicts near‐surface turbulence based on wind shear and buoyancy flux, that is, the extent of heating and cooling (Monin and Obukhov 1954; Wyngaard and Coté 1971; Grachev et al. 2015). MOST has been found to apply within water bodies over a range of sizes (Lombardo and Gregg 1989; Tedford et al. 2014; MacIntyre et al. 2018) but has not been tested in boreal lakes. These equations allow turbulence, as the rate of dissipation of turbulent kinetic energy, *ε*, to be estimated at the air–water interface, as needed for gas transfer velocities, and throughout the upper mixing layer to calculate the coefficient of eddy diffusivity (MacIntyre et al. 2018). The actual drivers of turbulence at the surface and in the upper mixing layer can be determined from the ratio of the shear and buoyancy terms, the Monin–Obukov length scale, *L*_MO_. At depths shallower than *L*_MO_ under cooling, the contribution from shear exceeds that from buoyancy flux. At a given depth *z*, for instance near the surface where gas exchange occurs, a time series of *z*/*L*_MO_ illustrates when shear is the driver, and law of the wall scaling applies, and when buoyancy flux augments turbulence production. Testing of these equations, which are based on readily measured variables such as wind speed, relative humidity, and air and surface water temperature, can be done using instrumentation that directly measures turbulence, such as acoustic Doppler velocimeters and microstructure profilers.

The equations for near‐surface turbulence and resultant gas exchange velocities can also be evaluated when chambers are used to compute flux (*F*) of dissolved gases. *F* is the product of the gas transfer velocity (*k*) and the concentration gradient across the thin layer on the water side of the air–water interface:

(1) $$F=k\;\left(C_{w}–C_{\mathrm{eq}}\right),$$

where *C*_w_ and *C*_eq_ are the actual concentration in the water near the air–water interface and the concentration in the water in equilibrium with the atmosphere, respectively. *k* can be obtained by inverting Eq. 1. Turbulence, as the rate of dissipation of turbulent kinetic energy (*ε*), is included when gas transfer velocities are computed using a surface renewal model (Zappa et al. 2007; MacIntyre et al. 2010; Wang et al. 2015):

(2) $$k=c_{1}\;{\left(\varepsilon \nu \right)}^{1/4}\;{\mathrm{Sc}}^{-n},$$

where *ν* is kinematic viscosity, *c*_1_ is a coefficient, Sc is the Schmidt number, and *n* is usually 0.5 for fluid interfaces (Csanady 2001; Zappa et al. 2007). For comparative purposes in freshwater, the gas transfer velocity is normalized to that for CO_2_ at 20°C for which the Schmidt number is 600 and is called *k*_600_. Tedford et al. (2014) provide new equations for turbulence based on near‐surface shear and buoyancy flux using results of microstructure profiling in a temperate lake. The surface renewal model is based on the concept that concentrations of dissolved gases are renewed at the air–water interface; the velocity of renewal events is captured by the Kolmogoroff velocity scale (*εν*)^1/4^ whose units are m s^−1^. The surface is renewed by upwelling events which cause divergence at the surface, that is, separation of parcels of water with the surface divergence quantified as *γ* = (*δu*/*δx* + *δv*/*δy*), where *u* and *v* are velocities in the *x* and *y* directions, respectively. Gas transfer velocities can be computed using the surface divergence model as:

(3) $$k=c_{2}\;{\left(<|\gamma |>\nu \right)}^{1/2}\;{\mathrm{Sc}}^{-n},$$

where *ν* is kinematic viscosity and *c*_2_ and *n* equal 0.5 (McKenna and McGillis 2004). While the frequency of near‐surface upwelling events has been linked to surface divergence, only a few field studies have attempted to evaluate whether the two methods would give similar estimates of gas transfer velocities (Wang et al. 2015). Although wind‐based models have routinely been used to compute *k* (Wanninkhof 1992; Cole and Caraco 1998), recent comparisons of the turbulence‐based surface renewal model and the wind‐based models indicate the latter may underestimate fluxes (Heiskanen et al. 2014; Mammarella et al. 2015; Czikowsky et al. 2018).

Scaling approaches have been developed to predict when turbulence will be induced at the base of the mixed layer and across the thermocline. Key dimensionless indices are the Wedderburn (*W*) number, and, for a lake as a whole, its integral from, the Lake number (*L*_*N*_) (Imberger and Patterson 1990; Horn et al. 2001). These depend on the extent of stratification, wind shear, and bathymetry. The Wedderburn number can be computed for diurnal or seasonal thermoclines. Wind pushes surface water downwind, depressing diurnal and/or seasonal thermoclines (Mortimer 1952, 1961; Imberger 1985). On relaxation of the wind, upwelling occurs driving surface water to the other end of the lake. Low values of *W* and *L*_*N*_ indicate appreciable upwelling may occur as well as shear across the thermocline that may induce mixing and deepening of the upper mixed layer (MacIntyre et al. 1999, 2009a; Horn et al. 2001). The rate of horizontal spreading of upwelled water depends onthickness of the stratified layer below the upper mixed layer and wind shear and is quantified by a horizontal dispersion coefficient (K_H_) (Monismith 1986). The extent of upwelling and downwelling of diurnal and seasonal thermoclines, and related vertical mixing and spreading in the horizontal, in small boreal lakes is not known. *L*_N_ or the ratio of the Monin–Obukhov length scale divided by mixing layer depth (*L*_MO_/*z*_AML_) may be able to predict the patchy mixing in the metalimnion observed when upper mixed layers are shallow (Antenucci and Imberger 2001). Even if vertical mixing across the thermocline is suppressed, wind‐driven circulation in the mixed layer may be critical for sustaining near‐surface turbulence once winds have ceased and for littoral‐pelagic exchange. The magnitude of cooling, that is negative buoyancy flux (*β*), relative to stratification quantified as the buoyancy frequency (*N*) may also be predictive of the extent to which dissolved gases would be entrained from the thermocline and mixed to the air–water interface. Scaling approaches, including MOST, *W*, *L*_*N*_, and *L*_MO_/*z*_AML_, allow determination of the processes causing mixing and transport, computation of coefficients required to compute fluxes, and are expected to improve accuracy in computing lake metabolism and greenhouse gas emissions.

The goal of our 5‐day study was to quantify the hydrodynamics of a small, highly stained boreal lake with a specific emphasis on measurements relevant to metabolism and emissions of climate forcing trace gases. Thermistor arrays across the lake illustrated the upwelling and downwelling within and below the upper mixed layer and provided a basis for assessing vertical and horizontal exchange. Frequency analysis of time series temperature data illustrated when turbulence increased in the upper thermocline. We related these results to Wedderburn and Lake numbers and the extent of cooling. We directly measured turbulence adjacent to the air–water interface and below using acoustic Doppler velocimetry and temperature‐gradient microstructure profiling. We compared these results with turbulence calculated from surface energy budgets using MOST and further used these data to estimate gas transfer velocities with the surface renewal model and to calculate the coefficient of eddy diffusivity. We measured surface temperatures with an infrared (IR) camera and computed near‐surface velocities based on sequential photographs taken at high speeds. These data allowed us to compute surface divergence and gas transfer velocities. In addition, we measured surface concentrations of CO_2_ and short‐term fluxes of CO_2_ with chambers and eddy covariance. These data allowed us to compare our physically based computation of gas transfer velocities with ones obtained using inverse methods based on empirical measurements. Our measurements allowed us to evaluate the accuracy of the equations for turbulence derived from MOST and surface divergence using IR methods, to improve wind‐based approaches to compute *k*, and to determine whether near‐surface turbulence was driven by shear or was augmented by buoyancy flux as winds decreased. As both *k* and *K*_*z*_ are essential for computing metabolism within lakes, we illustrate how their magnitudes change on time scales relevant to metabolic processes.

## *Site description and methods*

**Fig. 1:**
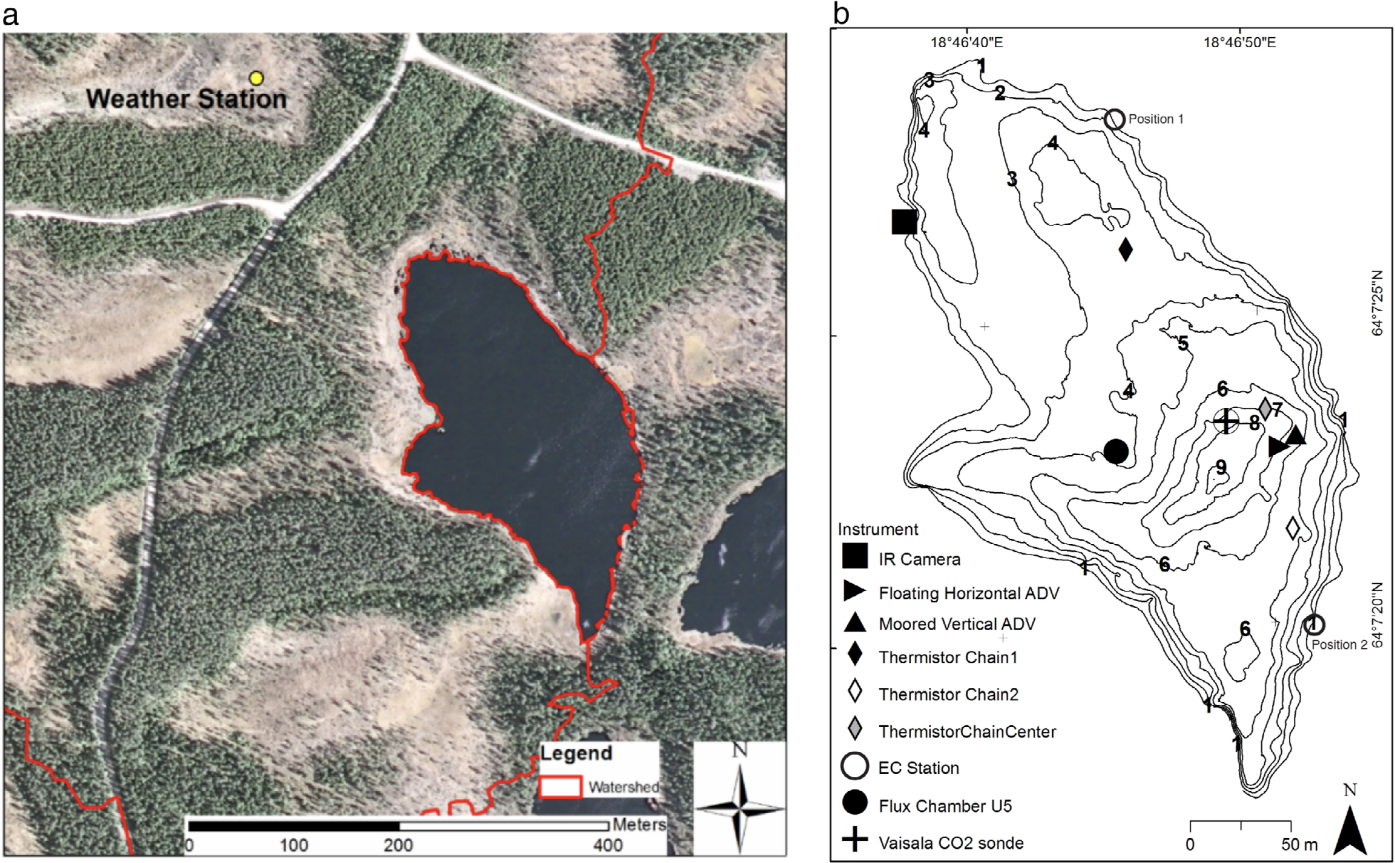
(**a**) Photograph of Övre Björntjärn showing surrounding forest, open land, and location of the meteorological station; (**b**) bathymetric map of Övre Björntjärn showing instrument locations. Numbers indicate contours in meters. The eddy covariance station (EC) was moved from position 1 to position 2 on the morning of doy 235 based on predicted changes in wind direction. SCAMP profiles were obtained to the east and west of the sonde platform and thus near the station where chambers were being deployed (U5).

The study was conducted in a small (surface area: 4.8 ha, maximum depth: 9 m, mean depth: 4 m) boreal lake, Övre Björntjärn, Sweden (64°7′25″N, 18°46′45″E) (Fig. 1a,b). Much of the northern basin is shallower than the mean depth. Dissolved organic carbon (DOC) is high (22 mg L^−1^), and pH is 4.0. The lake is surrounded by a Norwegian spruce, birch, and Scots pine forest; a mire and incoming stream are to the north and an outgoing stream is to the south (Fig. 1a). Additional details regarding the lake are provided in Klaus (2017). The lake was instrumented from doys 233 to 237, 20–24 August 2012.

### Meteorological instrumentation

A meteorological station was located 300 m northwest of the lake in a wetland that created a clearing in the forest (Fig. 1). This station provided continuous data and was the primary source of meteorological data for our analyses. Sensors measured wind speed and direction (Onset S‐WCA‐M003, 10 m above ground; air temperature (Onset S‐THB‐M002) and relative humidity (Onset S‐THB‐M002), both 1.5 m above ground), atmospheric pressure (Onset S‐BPB‐CM50), and photosynthetically available radiation (PAR, 400–700 nm, Onset S‐LIA‐M003). Data were averaged every 5 min. A net radiometer (Kipp & Zonen NR‐Lite) was deployed on the lake shore during the latter part of the experiment. Morphometry was obtained using a Lowrance HDS‐5 Gen2 echo sounder (Klaus 2017). We converted the measured PAR to shortwave using equations in Kalff (2002) and assumed an albedo of 3% throughout the day. Due to the brief deployment of the net radiometer, we let LW_net_ = −50 W m^−2^ as is typical for cloudy conditions (MacIntyre et al. 2009b). Light attenuation was computed following Beers Law using measurements from a LiCor underwater PAR sensor with cosine collector.

An eddy covariance (EC) system was installed on a 1.5‐m tripod situated on the shore of the lake and included one Gill Windmaster sonic anemometer (Gill Instruments Ltd, Lymington, Hampshire, UK) to measure the three wind components and sonic temperature, and a LiCor‐7500A (LiCor Inc., Lincoln, NE) open‐path gas analyzer to measure humidity and CO_2_. The instrument was initially on the northeastern shore and subsequently moved to the southeastern shore (Fig. 1b). A double rotation was performed on the sonic wind data. The signals were then detrended, despiked, and corrected for time lags. The EC fluxes were averaged over 30 min periods, and the Webb–Pearman–Leuning correction was applied in the postprocessing (Webb et al. 1980). To ensure a flux footprint over the lake, that is, that the winds were over the lake and not influenced by the adjacent terrestrial environment, data used in the analysis were constrained to wind speeds > 1 m s^−1^ and wind direction (WD) within the range 170° < WD < 210° for the initial deployment and 285° < WD < 350° for the second deployment. The final step in data quality control included a sorting of data based on a manual inspection of spectra and co‐spectra; data not following the spectral and co‐spectral theory were discarded. Sahlée et al. (2014) provide a detailed description of EC data analysis procedures. We applied the footprint model of Hsieh et al. (2000) and only accepted estimates of fluxes of CO_2_ when *L*_a_ < −4, where *L*_a_ is the Monin–Obukhov length scale in the atmosphere, defined below. For *L*_*a*_ = −4, the distance from the measuring point to the maximum contributing source area (Fp) = 8 m and 80% of the signal is obtained within 50 m of the EC station. For less negative values of *L*_a_, Fp becomes progressively smaller. For *L*_a_ = −10, 80% of the signal is obtained within 75 m of the station. Our criterion was typically met when *z*/*L*_a_ > − 0.5, where *z* is instrument height, as has also been applied elsewhere when accepting estimates of fluxes of CO_2_ (Heiskanen et al. 2014; Czikowsky et al. 2018). Due to the discontinuous EC data collection, the data from the weather station are the primary data used in analysis requiring the surface energy budget; the EC data are compared with results from the weather station to improve understanding of meteorology.

### Within lake instrumentation

Three thermistor arrays were deployed; two had all loggers on taut moorings from the surface downward whereas at the central one, the upper‐most loggers were suspended below a surface float and the deeper ones were on a taut‐line mooring with the subsurface float 0.27 m below the water surface. The arrays at the northern and southern stations were comprised of SeaBird 56 loggers sampling at 2 Hz. Loggers were 0.35, 0.65, 0.95, 2.0, and 3.0 m below the surface in the northern array and 0.35, 0.95, 2.0, and 4.0 m below the surface in the southern array. RBR 1050 loggers, sampling every 5 s, were located at 0.05, 0.35, 0.72, 1.01, 1.98, 2.95, 3.94, and 4.95 m below the surface at the mid‐lake station. All loggers have absolute accuracy of 0.002°C. We computed the depth where the temperature difference relative to the upper 0.05 m first reaches 0.02°C. The depth of the actively mixing layer, *z*_AML_, was operationally defined as the depth of the first thermistor immediately below that depth. Our operational criteria to quantify *z*_AML_ from time series temperature data are based on examination of microstructure data from a number of water bodies (e.g., Tedford et al. 2014) and takes into account, as we verified in this study, that the depth of near‐isothermy can be slightly deeper than the spacing of the thermistors or that near‐surface mixing sometimes extends into the stratified water immediately below a near‐isothermal layer. Data from the mid‐lake station were used in calculating *z*_AML_ and the metrics described below. The term upper mixed layer, or epilimnion, whose depth we identify based on the temperature jump at its base (minimally 0.5°C over 0.3 m in this study), refers to the weakly stratified layer above the thermocline. It can be subdivided into an actively mixing layer which is the region within it from the surface downward which is turbulent, a diurnal thermocline, and a subsurface layer (Imberger 1998). The vertical extent and magnitude of turbulence within these regions changes over diel cycles.

The three components of velocity were measured with a Nortek Vector acoustic Doppler velocimeter (ADV) oriented vertically with buoyancy provided by styrofoam floats arranged in a collar just below the transducer arms. Sampling was at 8 Hz, which in our experience causes less spiking than at higher frequencies (Umlauf and Arneborg 2009; Gålfalk et al. 2013), with each deployment lasting slightly longer than 2 days. The depth of the measurement volume, *z*_ADV_, was 0.15 m below the air–water interface for the first deployment and 0.25 m below for the second. Pitch and roll corrections were done using software provided by Nortek. Data were averaged in 10‐min blocks. Turbulence was quantified as *ε* following MacIntyre et al. (2018). To avoid the confounding influence of surface waves, we computed *ε* in the high wave number and low‐frequency portion of the power spectrum. We used the slowly varying advective flows associated with the low‐frequency flows to determine when the assumptions of Taylor's frozen field hypothesis, that the turbulent velocity fluctuations were smaller than the mean flow (Tennekes and Lumley 1972), were met, and we rejected results when they were not. Computing dissipation using the −5/3 law assumes that the turbulence is homogeneous and isotropic and that an inertial subrange exists (Tennekes and Lumley 1972; Thorpe 2007). The spectra for the *w* component decayed with a −5/3 slope as expected for homogeneous, isotropic turbulence and followed the Nasmyth universal spectrum (Tennekes and Lumley 1972; Oakey 1982). As is typical, the noise levels for the horizontal velocities were higher than for the vertical, and in this study, spectra for the *u* and *v* components were typically flat due to noise at frequencies lower than that of the surface waves. In consequence, we were unable to test for anisotropy as in MacIntyre et al. (2018) and accepted all dissipation rates for the *w* component that fit the Nasmyth universal spectrum and met the criteria of Taylor's hypothesis.

We also deployed an ADV horizontally but rejected those data as computed dissipation rates increased to values two orders of magnitude higher than those obtained with the vertically oriented instrument during periods when winds were shifting direction. In such cases, we assumed that vortices were being shed from the supporting frame which confounded the measurements. We additionally measured temperature with a fast‐response sensor located adjacent to the measuring volume of the ADV and logged it in the ADV recorder at the same rate as the velocities (8 Hz) to ensure that temperature and velocity were synchronized. We computed heat flux directly as <*T*′*w*′>,where *T*′ are the temperature fluctuations and *w*′ are the vertical velocity fluctuations.

### Temperature‐gradient microstructure profiling

Turbulence within the water column was measured with a temperature‐gradient microstructure profiler (SCAMP) used in rising mode. Details are provided in MacIntyre et al. (1999) and Tedford et al. (2014). The drag plate is designed such that by pointing the SCAMP in different directions on deployment, different locations are sampled. To further ensure that the instrument did not sample the same water mass, we moved a few meters horizontally along the boat's long anchor line after each deployment and moved to new locations after every few casts. Dissipation rates were calculated as in MacIntyre et al. (1999, 2009a) with goodness of fit criteria following Ruddick et al. (2000). Data were rejected when the mean absolute deviation >2.83 or when the likelihood ratio, which compares the fit of the data to a Batchelor spectrum and to a power law and takes the log of the ratio, was less than 1. These criteria are based on visual inspection of spectra on a number of profiles in which we assessed whether the goodness of fit tests were correctly rejecting poor fits and ensured that the data were clipped correctly at the top of the profile. Thorpe scales, that is, the size of instabilities indicative of turbulent regions, are presented as centered displacement scales.

The coefficient of eddy diffusivity was calculated during stratified conditions following Osborn (1980; Shih et al. 2005; Bouffard and Boegman 2013). *K*_*z*_ = Γ^.^ *ε* ^.^N^−2^, where *N* is buoyancy frequency and Γ is mixing efficiency (Osborn 1980). Under penetrative convection, energy for mixing produced by the surface buoyancy flux (*β*) is transported to the lower half of the actively mixing layer where some is used for mixing (*b*), some is dissipated, and some of the energy may subsequently be transported upward (Chou et al. 1986). The energy transported for mixing is at most half of the surface buoyancy flux. Using the definition of Γ in Wuest and Lorke (2003), *b*/*ε*, letting *b* = 0.5 *β*, and with dissipation rates in the lower half of the actively mixing layer ranging from 0.75 *β* to 0.45 *β* (Chou et al. 1986; Tedford et al. 2014), Γ ranges from 0.75 to 1. In our calculations, we let Γ = 0.8. Given that the steady‐state assumptions behind Osborn's (1980) approach may not be met for large Γ such as these, we address the uncertainty in these numbers by estimating *K*_*z*_ as *b*/*N* ^2^ (Wuest and Lorke 2003) for the lower half of the actively mixing layer, and we approximate *K*_*z*_ in the actively mixing layer, as *K*_*z*_ = *c*_4_^.^ *u l*, where *c*_4_ is of order 1, *u* is turbulent velocity scale, and *l* is depth of the actively mixing layer (Tennekes and Lumley 1972). Under convection *u* = w_*_, the turbulent velocity scale for cooling: w_*_ = (*βl*)^1/3^ and *β* is buoyancy flux defined below. We compute entrainment rate into the actively mixing layer as *dl/dt = β/l·N* ^2^ (Turner 1973). At the base of the actively mixing layer, where stratification is stable, shear from internal wave motions could energize a flux (Turner 1973), and the lower mixing efficiencies for stable stratification would apply.

### Measurements of CO_2_ concentrations and fluxes

Near‐surface concentrations of CO_2_ (*C*_*w*_) were obtained from headspace extraction using 1075 mL of water and 50 mL of air, correcting for the temperature dependence of gas–water partitioning in Henry's Law, and from equilibrated floating chambers equipped with CO_2_ sensors as described in Bastviken et al. (2015). The equilibrated headspace sample was transferred to a dry 50‐mL syringe and analyzed within 12 h on a cavity ring‐down spectrometer (Los Gatos Research model DLT 100 adapted for analysis of discrete sample injection). Parallel measurements with both methods yielded CO_2_ concentrations differing less than 5%. *C*_eq_ was calculated by Henry's law using air CO_2_ concentrations measured with the sensors or from syringe samples collected near the water surface and analyzed on the spectrometer.

The CO_2_ flux (*F*) was measured using five floating chambers (round plastic chambers with a volume of 5.4 liter and covering 0.062 m^2^; Natchimuthu et al. 2017) deployed for 15–40 min with concentrations measured 2–4 times per deployment. Chambers were deployed at approximately the same time as SCAMP profiling was conducted. CO_2_ samples were collected from chambers using syringes and the concentrations were analyzed on the cavity ring‐down spectrometer. The changes in ppm per time unit (Δppm/Δ*t*) derived from the linear slope of ppm CO_2_ vs. time, were converted to flux according to

(4) $$F=[\left(\text{Δppm}/\Delta t\right)\cdot P_{\mathrm{tot}}\cdot V/\left(R\cdot T\right)]\cdot^{}\left(1/A\right),$$

where *P*_tot_ is the total barometric pressure, *V* is the chamber volume, *R* is the common gas constant, *T* is the temperature, and *A* is the chamber area. Three cases with nonlinear data, that is, *r* ^2^ <0.9

### Calculation of the surface energy budget, dissipation rates, *W*, *L*_*N*_, *K*_*z*_, and gas transfer velocities from time series meteorological and temperature data

The surface energy budget was calculated using drag and mass transfer coefficients adjusted for atmospheric stability as in MacIntyre et al. (2002, 2014). Momentum and latent (LE) and sensible (SE) heat fluxes computed with these procedures differ by at most 10% from results obtained using COARE equations (Fairall et al. 1996; Tedford et al. 2014). On the two occasions when wind speeds were briefly below instrument threshold, we assumed wind speeds were 0.1 m s^−1^. The Monin–Obukhov length scale for the atmosphere, which indicates the stability of the atmosphere, may be thought of as a length scale that defines the importance of wind power relative to buoyancy flux. It is computed as

(5) $$L_{a}=\left(-\rho \;{u_{*}}^{3}T_{v}\right)/\left(\kappa^{.}g^{.}\left(\mathrm{SE}/c_{\mathrm{pa}}+0.61\left({T_{z}}^{.}\mathrm{LE}/L_{v}\right)\right)\right),$$

where u_*_ is the air friction velocity computed from shear stress *τ* as ρu_*_^2^ = $\rho C_{d}U_{z}^{2}$ = *τ*, *ρ* is density of air, *g* is gravity, *κ* is the von Karman constant ~ 0.4, *U*_*z*_ is wind speed measured at height *z*, *C*_d_ is the drag coefficient at instrument height *z*, *T*_v_ is virtual air temperature at height where air temperature *T*_*z*_ is measured in degrees Kelvin, *T*_v_ = *T*_z_^.^[1 + 0.61*q*_*z*_] ; *q*_*z*_ is saturated specific humidity (*see* MacIntyre et al. 2014); *c*_pa_ is specific heat of air; *L*_v_ is latent heat of vaporization, and SE and LE are sensible and latent heat flux, respectively. The drag coefficient varies with stability of the atmosphere, being higher when it is unstable and lower when stable for the same wind speed, thus the process of computing *L*_a_, *C*_d_ as well as the mass transfer coefficients for heat and water vapor, u_*_, LE, and SE is done iteratively (Hicks 1975). The Monin–Obukhov length scale on the water side is *L*_MO_ = $u_{*w}^{3}/\kappa \beta$, where u_*w_ is the water friction velocity computed assuming that shear stress is equal on the two sides of the air–water interface, $\rho_{w}u_{*w}^{2}=\rho u_{*}^{2}$, and *β* is buoyancy flux. *β* = *g*α*H*_eff_/*c*_pw_ *ρ*_w_ where *g* is gravity, *α* is the thermal expansion coefficient, *c*_pw_ is the specific heat of water, and *H*_eff_ is heat flux in the surface layer. *H*_eff_ is estimated as: *H*_eff_ = SW_net_ – SW_S_ + LE + SE + LW_net_, where SW_net_ is net incoming shortwave, SW_S_ is the SW leaving the surface layer at its base, and LW_net_ is net long wave radiation. *L*_MO_ indicates the relative magnitude of turbulence production from wind and from buoyancy flux (Csanady 2001). When positive (negative), the upper water column is heating (cooling); the magnitude indicates the extent of the surface layer influenced by wind. The ratio *L*_MO_/*z*_AML_ indicates the fraction of the surface layer in which shear production of turbulence dominates over buoyancy flux (Imberger 1985). For example, under cooling, if |*L*_MO_/*z*_AML_| = 0.1, only the very near surface is energized by wind, whereas if |*L*_MO_/*z*_AML_| = 1, the full surface layer is being mixed by wind. The coefficient of eddy diffusivity was computed at half‐day intervals below the upper meter using the heat budget method (Jassby and Powell 1975).

**Fig. 2:**
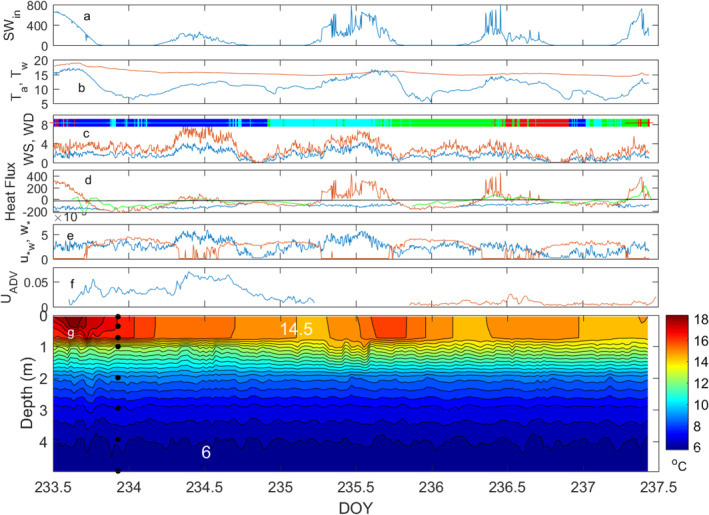
Time series of (**a**) incoming shortwave radiation, W m^−2^; (**b**) air (blue) and surface water temperature (brown), °C; (**c**) measured wind speed (blue) and maximum wind speed (brown) in m s^−1^ with wind direction overlaid by quadrant (north, green; south, blue; east, red; west, cyan); (**d**) surface (blue) and effective (brown) heat fluxes and hourly averaged heat flux (green) measured by the paired rapid response thermistor and ADV at 0.15 m (first deployment) and at 0.25 m (second deployment) in W m^−2^; and (**e**) u_*w_ (blue) and w_*_ (brown) in m s^−1^; (**f**) 10‐min averaged current speeds measured by the ADV with measurement volume at 0.15 m (blue) and 0.25 m (brown) in m s^−1^; (**g**) temperature contours averaged over 25 min; black dots are depths of thermistors. Data in panels (**a**) through (**e**) are 5‐min averages.

**Fig. 3:**
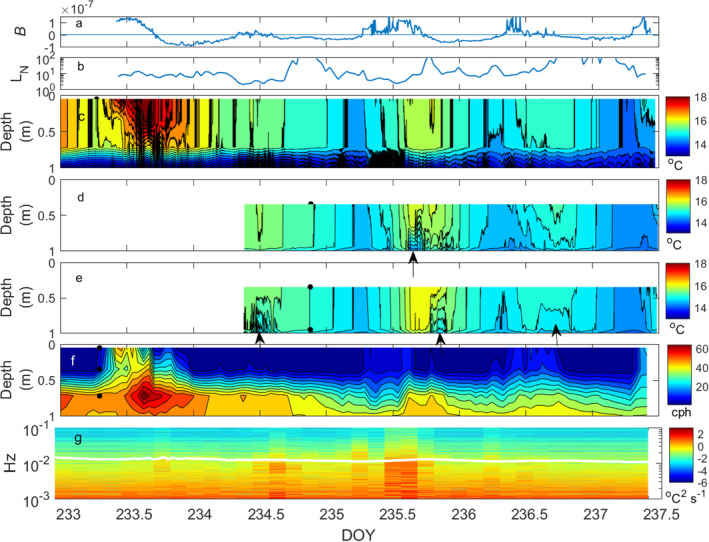
Time series of (**a**) buoyancy flux (*β*) in m^2^ s^−3^; (**b**) 45‐min averaged Lake number (*L*_*N*_); (**c**–**e**) 1‐min averaged temperature contours within the upper meter with (**c**) at the center station, (**d**) the north station, and (**e**) the south station; (**f**) hourly averaged buoyancy frequency (*N*) in cycles per hour (cph) in the upper meter at center station. Contours are based on data from all measurement depths with only the upper meter shown. (**g**) Spectrogram of temperature fluctuations at 1‐m depth at the center station (°C^2^ s^−1^) with *N* (cps) in white overlay. Segments were 1.2 h with data at 5‐s intervals; a Hamming window was applied to the nonoverlapping segments. Fluctuations are considered turbulent when the amplitude increases at frequencies above *N*. Arrows in panels (**d**) and (**e**) mark upwelling (*see* also Figs. 4, S7).

Wind speed and direction and calculated LE and SE for the weather station are similar to measurements with the EC system except that LE and SE from the EC system were sometimes more positive at night and more negative in the day (Fig. S1). Computed heat fluxes were similar with the main discrepancy being heat losses were up to − 50 W m^−2^ larger at night at the weather station (Fig. S2). Computed buoyancy fluxes only differed by a factor of two at such times (data not shown); thus the effects on computed *ε* were small. Wind speeds were corrected to neutral condition at 10 m height (WS_10_) taking into account atmospheric stability (Fig. S3) (Smith 1988), and these corrected values are used in the analyses of *ε* and *k*_600_ relative to wind speed. Measured wind speeds are referred to at other times (e.g., Figs. 2, 3). On days when winds at the weather station were light (< 3 m s^−1^), wind speeds were often higher over the lake than over the wetland (Figs. S1, S3).

We computed the depth dependent *ε*_*z*_ within the surface mixing layer from the meteorological data from the weather and EC stations and within lake thermistors following the similarity scaling of Tedford et al. (2014). During cooling, *ε*_*z*_ = 0.56 $u_{*w}^{3}/\mathrm{\kappa z}$ + 0.77 *β*_,_ and during heating *ε*_*z*_ = 0.6 $u_{*w}^{3}/\mathrm{\kappa z}$, where u_*w_ is the water friction velocity computed from wind shear stress *τ* = *ρC*_d_*U*_*z*_^2^ = $\rho_{w}u_{*w}^{2}$, *U*_*z*_ is wind speed at height *z*_*a*_, *C*_d_ is the drag coefficient at height *z*_*a*_, *ρ* and *ρ*_w_ are density of air and water, respectively, *κ* is von Karman's constant, *z* is depth, and *β* is surface buoyancy flux (Tedford et al. 2014). The equation *ε*_*z*_ = $c\cdot u_{*w}^{3}/\mathrm{\kappa z}$, in which dissipation rates are independent of *β* and *c* is an empirically determined coefficient, implies law of the wall scaling. We let *z* = 0.15 cm when calculating dissipation rates using the similarity scaling for the first ADV deployment and 0.25 m in the second to coincide with the depth of the measurement volume of the ADV. We also computed *ε* as a function of *β* under cooling as *ε*_*β*_ = 0.77 *β*_._

Buoyancy frequency, which indicates the strength of stratification, was computed as *N* = (*g*/*ρ*_w_^.^ *dρ*_w_/*dz*)^1/2^, where *g* is gravity, *ρ*_w_ is density of water computed following Chen and Millero (1977) with results as in newer equations (IOS et al. 2010). We computed internal wave periods for the density stratification in the lake following Gill (1982). We computed Lake numbers as in MacIntyre et al. (1999) and Wedderburn numbers when diurnal thermoclines were present as *W* = (*g*/*ρ*_w_) Δ*ρ h* ^2^/ $u_{*w}^{2}$. *L*, where g is gravity, Δ*ρ* is the density difference across the diurnal thermocline, *h* is the depth of the diurnal thermocline, and *L* is the length of the lake in the direction of the wind.

We computed *k*_600_ using the surface renewal model (Eq. 2) using dissipation rates from the ADV, the SCAMP, and from the similarity scaling following Tedford et al. (2014) and with coefficients *c*_1_ and *n* equal to 0.5. We also computed *k*_600_ using inverse procedures based on CO_2_ flux and surface water concentration measurements according to Eq. 1 rewritten as *k* = *F*/(*C*_w_ – *C*_eq_).

### IR camera system

*k*_600_ was also calculated using an IR camera system on the northwestern shore (Fig. 1a) (Gålfalk et al. 2013). Gas transfer velocities were calculated from *γ*, surface divergence, computed from maps of velocity fields across each image using Eq. 3 with *c*_2_ and *n* both equal to 0.5 (Fig. S4). The thermal IR camera (Cedip Titanium 520) is electrically cooled to 77°K, has a 3.7–5.1‐μm band‐pass filter, and was placed on a tripod 1.8 m above the water surface with a 10°–30° angle normal to the surface to minimize reflected IR light (emissivity close to 1). The field of view was 0.40 m × 0.37 m. Images were acquired at 100 Hz for 60 s for each *k*‐measurement, using a resolution of 320 × 256 pixels, and thermal structures were tracked in postprocessing to make velocity maps used to calculate the average divergence during each time frame. As the *k*‐model is based on surface divergence, the average water velocity does not affect the calculations. On the first day of the study, winds were southerly and the fetch extended the length of the lake. During subsequent measurements, winds were northerly such that the water surface near the camera deployment was sheltered.

## *Results*

Our goal is to describe the hydrodynamics of the small lake to inform studies of lake metabolism and greenhouse gas evasion. We first describe changes in thermal structure as a result of changing meteorology, wind‐induced currents, and internal wave dynamics as they moderate inshore–offshore exchange and vertical fluxes. We then illustrate mixing dynamics with time series profiles of temperature‐gradient microstructure data. Lastly, we quantify near‐surface turbulence using temperature‐gradient microstructure data and data from an acoustic Doppler velocimeter, and we evaluate how well a recently derived similarity scaling for lakes predicts near‐surface turbulence. We additionally contrast gas transfer velocities obtained from the turbulence measurements incorporated in the surface renewal model with those obtained with inverse procedures using chambers and with the surface divergence model to evaluate the differing models, address within lake variability in gas transfer velocities, and illustrate relations with wind speed.

### Meteorology and thermal structure

The upper mixed layer was shallow, ~ 0.75 m, and with the lake's high diffuse attenuation coefficient, 2 m^−1^ and small size, diurnal thermoclines formed within it during heating periods with light winds (Figs. 2, 3). Temperatures were above 14°C in the mixed layer and 4°C cooler by 2 m. With this strong temperature stratification, *N* ranged from 60 to 40 cycles per hour (cph) at the base of the mixed layer (Fig. 3). Variability in meteorological conditions led to changes in temperature in the upper mixed layer and slight warming of the upper thermocline which contributed to the decrease in stratification between 0.5 and 1 m (Figs. 2, 3). Temperatures below 2 m were largely unchanged.

**Fig. 4:**
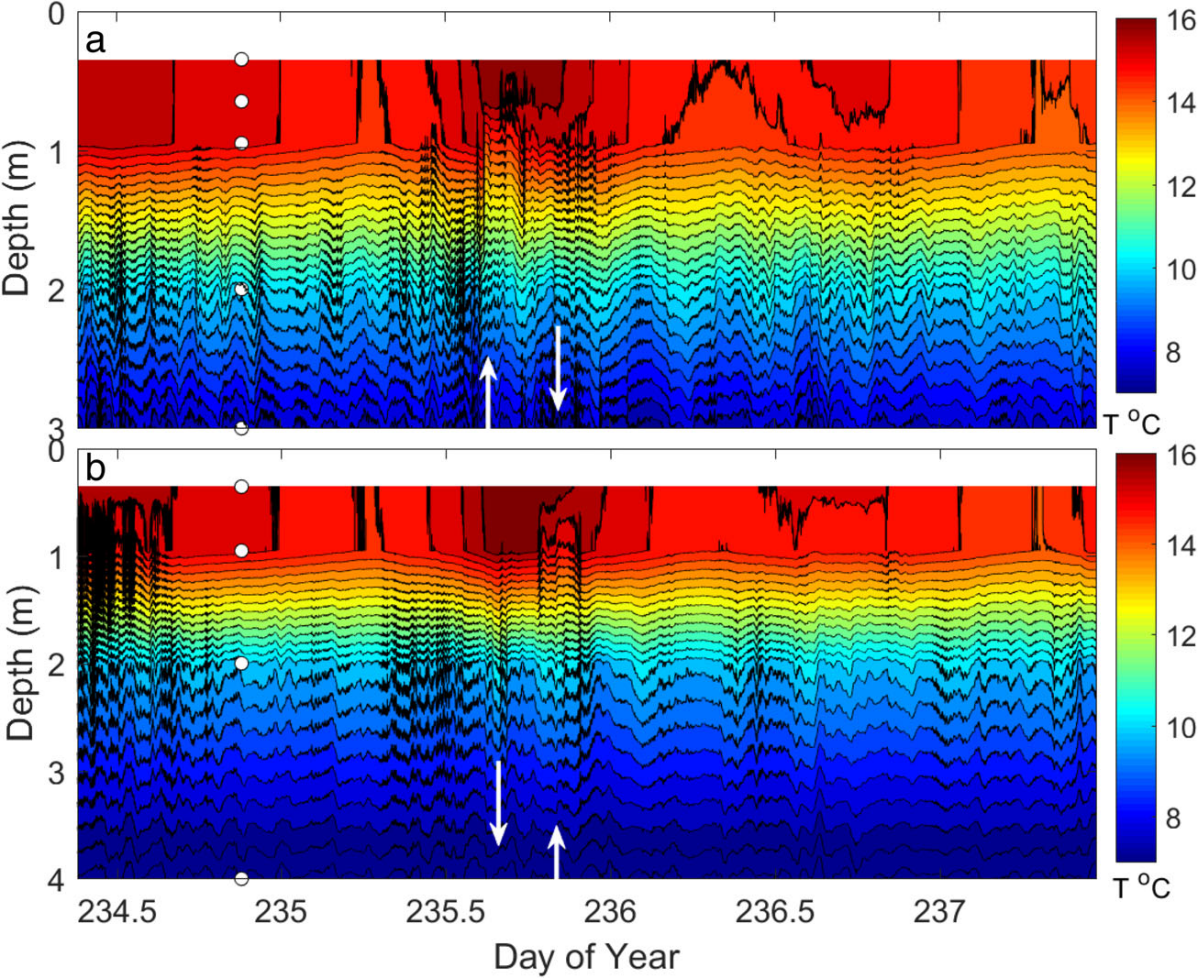
Time series temperatures as 5‐s averages at (**a**) northern and (**b**) southern temperature arrays with depths chosen to show the upper mixed layer and upper part of the metalimnion. Steep fronted upwelling of water from the upper thermocline into the mixed layer (up arrow, panel (**a**), doy 235) in response to southerly winds and corresponding thermocline compression to the south (down arrow, panel b, doy 235). On relaxation of the wind, the thermocline compressed to the north (down arrow) and a second vertical‐mode expansion occurred to the south (up arrow).

Sunny cloudless skies, air temperature of ~ 15°C, and light winds, ~ 2 m s^−1^ on the first day led to appreciable heat flux into the upper water column and formation of a diurnal thermocline (Figs. 2, S5). The following day, with extensive cloud cover, lower air temperatures, and southerly winds with gusts up to 8 m s^−1^, marked the transition to the later period with intermittent cloud cover, somewhat warmer air temperatures, and low‐to‐moderate winds. These differences led to differences in the flux of heat into the upper water column in the day and resultant stratification. Temperatures decreased on doy 234; warming occurred on doy 235 despite higher winds, and warming was less on the following days (Figs. 2, 3). Weak stratification developed within the upper mixed layer on doys 235–237 and was more persistent on doy 237 (Fig. S6). Air temperature was colder than surface water temperature except briefly on the afternoon of doy 235. Consequently, the atmosphere was unstable (*L*_a_ < 0), except for the one brief period on doy 235. Hence, momentum transfer to the water surface was augmented relative to a neutral atmosphere. Current speeds increased with wind speed, with those on doy 234 reaching 0.06 m s^−1^ (Fig. 2f). They persisted for at least an hour and a half after the wind dropped, indicating the potential for near‐surface shear and turbulence production after winds ceased. Heat flux computed as <*w*′*T*′> and the surface energy budget were similar (Fig. 2d). Values were slightly higher with <*w*′*T*′> than the surface energy budget when currents were measureable after the wind ceased and indicate heat flux from advection (Fig. 2d,f). Afternoon winds energized internal waves in the thermocline and hypolimnion (Figs. 4, S7).

Variations in the magnitude of buoyancy flux (*β*), and the effective heat flux from which it is calculated, and wind speed determine the drivers for turbulence in the actively mixing layer (Figs. 2d,e, 3a). During the day, when *β* was positive, near‐surface shear drove turbulence. The turbulent velocity scale from wind, u_*w_, reached 0.006 m s^−1^ on windy afternoons and decreased at night. At night and intermittently on the cloudy days, *β* was negative such that the surface layer cooled. The surface heat fluxes, that is the sum of LE, SE, and LW_net_, were −200 W m^−2^ in the initial clear sky period and subsequently were ~ −100 W m^−2^ with the lowest values at night when winds ceased (Fig. 2d). The turbulent velocity scale from heat loss, w_*_ = (*β* ^.^ *z*_AML_)^1/3^, had maxima of 0.004 m s^−1^ at night and tended to exceed u_*w_ (Fig. 2f). w_*_ vanished during heating but occasionally was nonzero during days with variable cloud cover and resultant intermittent cooling. In the day, near‐surface mixing was driven by wind with some contribution from cooling. At night and occasionally on doys 234 and 236, with nonzero values of u_*w_ and w_*_, both shear and heat loss are expected to cause near‐surface turbulence with shear production dominating at depths above *L*_MO_ and cooling below. The persistent currents when the wind ceased indicated some shear could be maintained to cause mixing, as on early evening doy 234 (Fig. 2f).

**Fig. 5:**
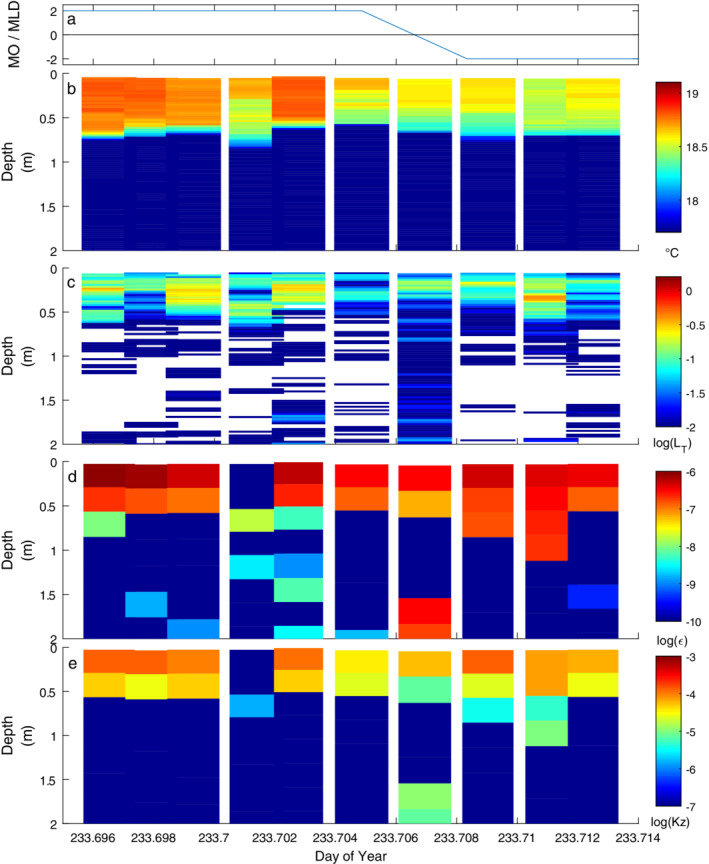
Time series of (**a**) *z*_MO_/*z*_AML_ and temperature‐gradient microstructure profiles of (**b**) temperature (°C), logarithm of (**c**) centered overturn scales (*L*_*T*_) (m), (**d**) rate of dissipation of turbulent kinetic energy (*ε*) (m^2^ s^−3^), and (**e**) coefficient of eddy diffusivity (*K*_*z*_) (m^2^ s^−1^) on doy 233 (21 August) with winds initially light under heating. The absence of overturning is indicated by white patches in profiles of *L*_*T*_.

**Fig. 6:**
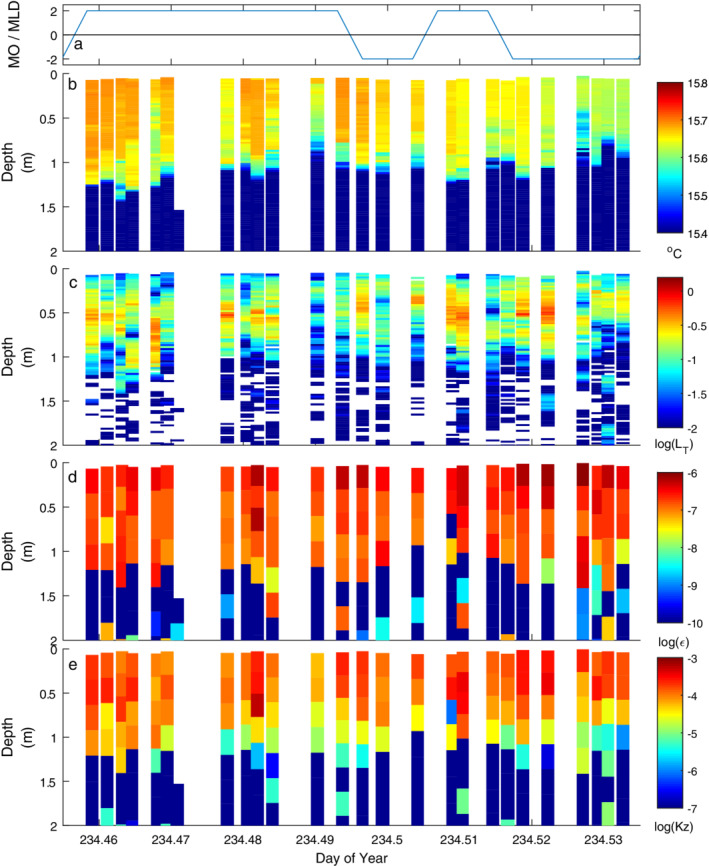
As for Fig. 5 on doy 234 with highest winds of the experiment.

**Fig. 7:**
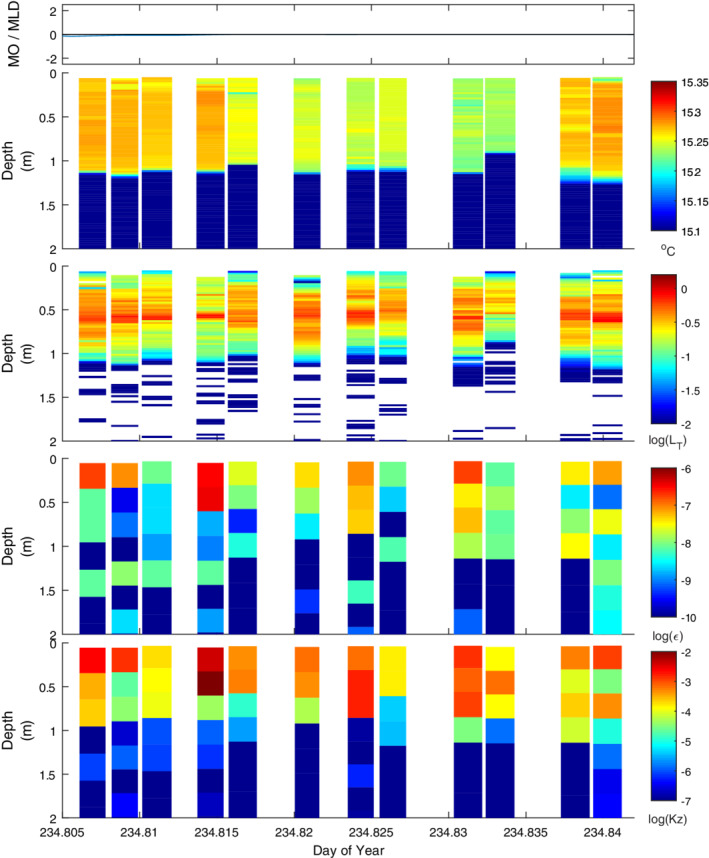
As for Fig. 5 but under cooling on evening doy 234. Winds dropped to anemometer threshold. Note change in scale for *K*_*z*_ relative to the other figures showing microstructure data.

**Fig. 8:**
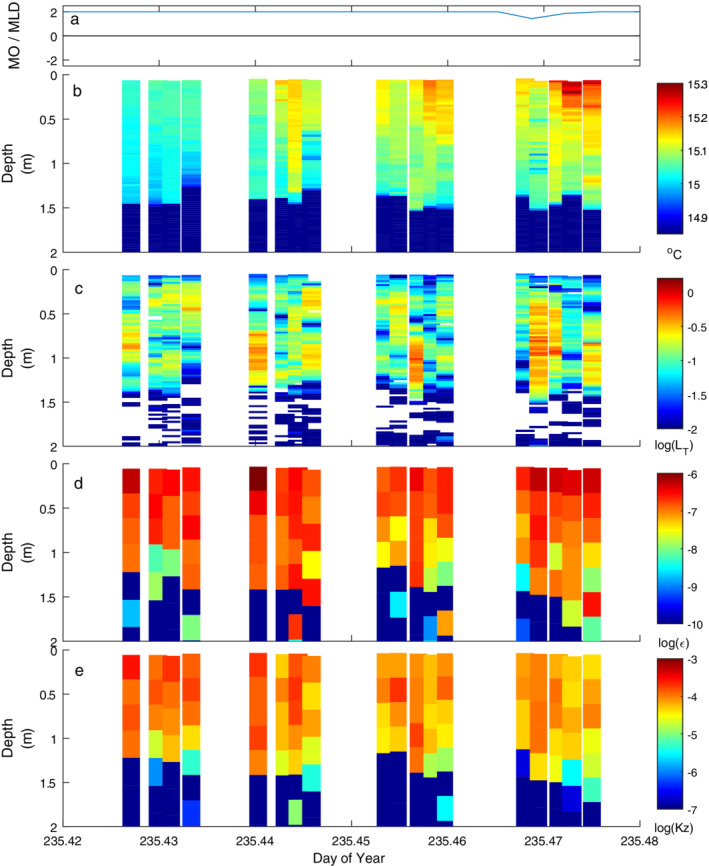
As for Fig. 5 with heating under moderate winds.

**Fig. 9:**
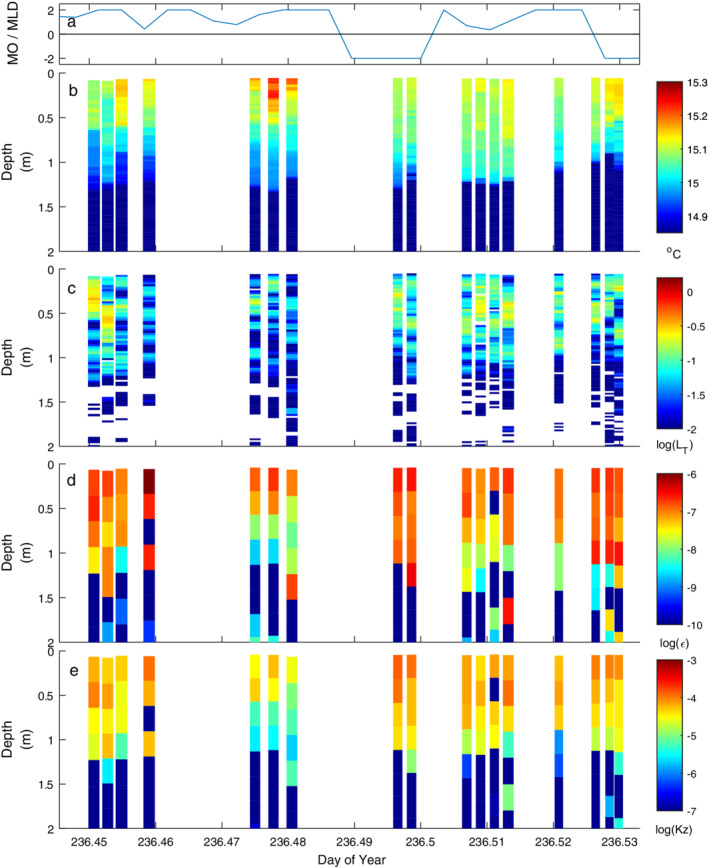
As for Fig. 5 with light winds and alternation between heating and cooling.

**Fig. 10:**
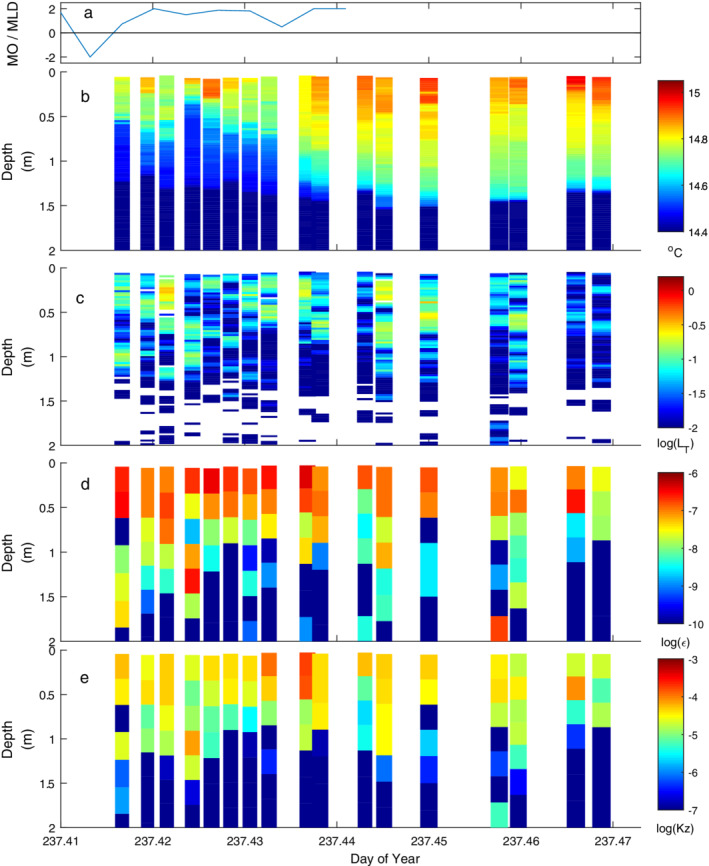
As for Fig. 5 and illustrating sensitivity of near‐surface temperature and *ε* to slight changes in wind speed and direction and consequent upwelling and downwelling of isotherms within the upper mixed layer (Fig. S6).

The ratio of the Monin–Obukhov length scale on the water side to the depth of the actively mixing layer, *L*_MO_/*z*_AML_, indicates the fraction of the mixed layer being mixed by wind with its sign indicating heating or cooling conditions (Imberger 1985). During the days with moderate winds when we conducted microstructure profiling, *L*_MO_/*z*_AML_ exceeded 1 regardless of the sign of *β*, indicating the actively mixing layer was fully energized by wind (Figs. 5, 6, 8). *L*_MO_/*z*_AML_ approached 0 under the lightest winds under cooling, indicating that shear would drive turbulence production at depths above *L*_MO_. However, when there was no wind at night, *L*_MO_ vanished, and near‐surface turbulence would be produced by cooling once residual currents ceased (Figs. 2f, 7). *L*_MO_/*z*_AML_ approached 0 under the lightest winds during heating (Figs. 9, 10). At such times, the ratio *z*/*L*_MO_ intermittently exceeded 0.1, and diurnal thermoclines formed (Figs. 3, S8). Buoyancy flux begins to contribute to enhanced shear and near‐surface turbulence when *z*/*L*_MO_ > 0.1 (Grachev et al. 2013, 2015; Tedford et al. 2014). Our ADV deployment and our microstructure profiling just missed this regime on doy 233 but, as will be discussed in the section on microstructure profiling, we captured it late morning doy 237 (Fig. 10).

### Mixing at the base of the mixed layer

Both cooling and wind contributed to mixing at the base of the mixed layer and the resultant decrease in stratification (Fig. 3). Wedderburn numbers across the diurnal thermoclines ranged from 0.1 to 1 on doys 233, 236, and 237. The expected upwelling and downwelling of the diurnal thermoclines is evident in Figs. 3c, S5, and S6 and would have led to cross‐lake differences in temperature and shear at the base of the mixed layer (Imberger 1985; Monismith 1986). Lake numbers decreased to values near 2 on doys 234 and 235. On these 2 days, diurnal mixed layers were weak and short lived, and, on doy 235, incoming heat was mixed throughout the mixed layer. The wind induced upwelling and downwelling at the base of the mixed layer is evident on all the days with three thermistor arrays with cool water upwelling upwind (Fig. 3c–e).

Mixing at the base of the mixed layer or top of the thermocline is identified by the decreases in *N* and by spectral energy from temperature fluctuations increasing at frequencies above *N* (Fig. 3f,g). The increases in spectral energy were most pronounced at 1 m on doys 234 and 235 as the Lake number dropped to 2 and are also evident on day 233 when it dropped to 6. Temperature fluctuations at frequencies indicative of mixing were also evident at night. Temperature only increased at 1‐m depth from the morning of doy 234 through the afternoon of doy 235, when *N* weakened at the top of the thermocline (Fig. 3f). Cloudy periods with winds sufficient for *L*_N_ to decrease toward 1 are critical for fluxes between the upper mixed layer and upper thermocline.

### Internal waves, horizontal transport, and mixing

The Lake number dropped to low values, 2–5, during windy periods, indicating that the wind was of sufficient magnitude that surface currents flowed downwind causing the thermocline to downwell (Figs. 3, 4, S7). Upwelling occurred on relaxation of the wind as currents reversed, and *L*_*N*_ increased above 10. Upwelling is evident to the south with northerly winds mid‐day 234 and to the north mid‐day on doy 235 after the winds shifted from westerly to northerly. When the wind relaxed at doy 235.75, the thermocline upwelled to the south. The sudden increases in temperature at the middle and northern stations at that time are indicative of northward flow of warm water from the south. *β* was negative at the time, so the warming is further evidence of northerly transport on cessation of the wind. These observations of wind induced advection are also supported by the measured heat flux <*w*'*T*'> being larger than that computed by the surface energy budget (Fig. 2d).

Horizontal advection was evident when Lake numbers were higher, a diurnal mixed layer formed, and Wedderburn numbers ranged from 0.1 to 0.5 (Fig. 3). Water temperatures were warmer to the south after midnight on doy 236, and once heating began, the mixed layer was deeper to the south than to the north, indicating northerly winds transported warmer water south. Wind speeds decreased and shifted from northerly to northeasterly mid‐day 236. As a result, mixed layer depth decreased to the south, temperatures did not decrease mid‐lake despite the onset of cooling, and near‐surface temperatures increased to the north by doy 236.7. These observations indicate northerly flow of warm water. With *W* across the diurnal thermocline less than 1 and *L*_*N*_ = 10, the increase in energy in temperature fluctuations at 1‐m depth was muted. Thus, while the upwelling and downwelling of the diurnal thermocline indicated cross‐basin transports, exchange across the thermocline was less than on the preceding 2 days when *L*_*N*_ decreased to lower values.

Second vertical‐mode internal waves formed when winds were high enough to cause *L*_*N*_ to fall into the range from 2 to 5, when winds relaxed, and when winds shifted direction (Figs. 4, S7). Thus, the upwelling and downwelling at the base of the mixed layer described above are manifested throughout the stratified water column. Cross‐basin transport would result as the thermocline alternately expanded and contracted at opposite sides of the lake. Following Gill (1982), the calculated periods for first and second vertical‐mode internal waves are 3 and 8.4 h on the longer north–south axis of the lake and 1.5–4.2 h on the 200 m east–west axis. The second vertical‐mode wave which initiated when the northerly winds decreased at 235.6 had a period of ~ 6 h. (Figs. 4, S7). It is reasonable to assume it is a seiche as predicted internal wave periods are modified by bathymetry (Fricker and Nepf 2000). High‐frequency temperature fluctuations occurred at the top of the thermocline and within the hypolimnion when upwelling from these waves was accentuated, particularly when *L*_*N*_ ~ = 2. The spectrogram analysis indicates that those at the top of the thermocline were turbulent (Figs. 3c,g). Upwelling at the northern and southern stations was also accompanied by high‐frequency temperature fluctuations. Microstructure data indicated that the intrusions of cooler water were turbulent (Figs. 6, 8). Thus, the wind‐driven internal wave motions, while not causing large amplitude movements of the thermocline as observed in larger lakes, did cause upwelling of the upper thermocline to shallower depths and cross lake advection. High‐frequency temperature fluctuations indicative of mixing occurred.

Eddy diffusivities computed following Jassby and Powell (1975) for half day time steps at depths below 1 m were less than 10^−6^ m^2^ s^−1^, indicating limited vertical transport below the mixed layer.

### Turbulence within the water column from microstructure profiling

The time series profiles with the SCAMP show the considerable variability in temperature and extent and variability of turbulence in the upper mixed layer in response to differing meteorological conditions (Figs. 5, 6, 7, 8, 9, 10). Turbulence was suppressed in the thermocline. Profiles were obtained either near mid‐day, with |*L*_MO_/*z*_AML_| typically greater than 2 indicating the wind was fully energizing the actively mixing layer and additionally, depths below it, or at night with *L*_MO_/*z*_AML_ equal to 0 as winds had dropped below the anemometer's threshold. Turbulent eddies, also called overturns, and identified by the extent of unstable regions within the temperature profile, were found throughout the mixed layer. They were 0.05–0.3 m in vertical extent during the day whereas they ranged from 0.3 m to 1 m at night. Overturns below the mixed layer tended to be less than a few centimeters. Dissipation rates were elevated throughout the mixed layer when |*L*_MO_/*z*_AML_ | > 2. They were highest near the surface, reaching 10^−6^ m^2^ s^−3^, and decreased with depth in the mixed layer, as expected when shear dominates turbulence production and follows law of the wall scaling. Higher values occurred under windier conditions (Figs. 5, 6, 7, 8, 9, 10). Below the mixed layer, both *ε* and *K*_*z*_, which is calculated from *ε*, tended to be below 10^−8^ and 10^−6^ m^2^ s^−1^, respectively. In the following, the turbulence is described for each day and context provided with respect to changes in thermal structure, values of *z*/*L*_MO_, *L*_*N*_, conditions during cooling, and when intrusions of cooler water occurred due to upwelling from the upper thermocline.

As a result of heating and low winds on doy 233, the water column was linearly stratified to the surface from late morning until early afternoon; *L*_MO_/*z*_AML_ intermittently approached 0, and *z*/*L*_MO_, where *z* is measurement depth of the ADV, exceeded 0.2 and occasionally 1 (Figs. 5, S8). The increase in wind just before we sampled depressed the diurnal thermocline such that the actively mixing layer was 0.5 m deep (Fig. S6), |*L*_MO_/*z*_AML_| > 1 indicating it was fully wind mixed, and *z*/*L*_MO_ < 0.1 (Fig. S8). Dissipation rates were elevated within the actively mixing layer, and *ε* near the surface ranged from 10^−7^ to 10^−6^ m^2^ s^−3^ (Fig. 5). Below the actively mixing layer, *ε* was at least an order of magnitude lower. The profiling captured the abrupt downwelling of the actively mixing layer at doy ~ 233.71, which resulted from subtle changes in wind speed and direction (Fig. S5) as well as that associated with the second vertical‐mode expansions and contractions deeper in the water column (doy 233.707, 1.75–2 m).

With the increased winds on doy 234, the upper mixed layer had downwelled at the central station. With *L*_MO_/*z*_AML_ > 1, the upper mixed layer was fully energized and overturns ranged in size from 0.1 to 0.4 m (Fig. 6). Dissipation rates at the surface had values similar to the those the previous day and decreased immediately below (Fig. 6d). However, they often increased at the base of the mixed layer and upper thermocline as expected with an increase in shear when *L*_*N*_ drops to low values. The increases in *ε* at the base of the mixed layer provide further support that the increases in energy in temperature fluctuations at frequencies above *N* were indicative of turbulence (Fig. 3g).

Dissipation rates were variable at night on doy 234 when winds were negligible and a light rain was falling, and *ε* was no longer consistently highest at the surface (Fig. 7). *L*_MO_/*z*_AML_ initially approached and then equaled 0, as would occur with no wind. Buoyancy flux was ~ 4 × 10^−8^ m^2^ s^−3^ (Fig. 3a), and surface values of *ε* were often of that magnitude, indicating the turbulence was induced by convection. Higher values either occurred near the onset of sampling when there was a light wind or were associated with overflows of warmer or cooler water which would have contributed to near‐surface shear.

Winds were unsteady and the buoyancy flux intermittently positive when observations were made on doy 235 (Fig. 8). Winds were sufficient for *L*_*N*_ to decrease to 2 and were primarily westerly with occasional northward gusts (Figs. 2, 3). With the variable winds, the mixed layer alternately downwelled and upwelled. With net positive buoyancy flux, heat was mixed downward and the stratification increased in the mixed layer (Figs. 3f, 8). The range of dissipation rates at the surface was similar to that on other windy days but the depth to which *ε* > 10^−7^ m^2^ s^−3^ varied from 0.5 to 1.5 m (Fig. 8d). The largest overturns tended to be centered at 1‐m depth (Fig. 8c), and the highly resolved temperature data showed intrusions were prevalent at the base of the mixed layer (Fig. 8b). Their structure, with interleaving cool and warm water, is indicative of Kelvin–Helmholtz billows. Due to the resulting mixing, dissipation rates were of order 10^−7^ m^2^ s^−3^. Hence, these features support the inference from *L*_*N*_ and wind direction that the thermocline had tilted along an east to west axis and shear was enhanced such that overturning occurred at the base of the mixed layer. They also support our inference of mixing when temperature fluctuations were energetic at frequencies above *N* (Figs. 2, 3, 4, S7).

Winds were lighter around noon on doys 236 and 237 when we profiled (Figs. 9, 10). The ratio *z*/*L*_MO_ exceeded 0.1 at times during our sampling (Fig. S8). On doy 236, *L*_MO_/*z*_AML_ frequently changed sign and intermittently was less than 1. While *ε* was highest at the surface, the depth to which values exceeded 10^−7^ m^2^ s^−3^ varied. On doy 237, prior to our measurements, the upper 0.3 m was linearly stratified, that is, it was entirely a diurnal thermocline (Fig. S6). With an increase in wind speed, cooler water upwelled in the lower 0.4 m of the upper mixed layer. As the winds tapered, a second vertical‐mode response occurred within the 0.7‐m upper mixed layer. Water from 0.25 m upwelled and that at 0.35 m downwelled. These motions imply horizontal movement of water to the central thermistor array and lateral displacement of the warmer near‐surface water. The first eight microstructure profiles captured the dynamics of the second vertical‐mode response. The frequent 0.1 m overturning scales in the upper 0.5 m co‐occurred with isotherm displacements of similar amplitude. Beginning at 237.435, wind speed decreased. Warmer water flowed back to the site and the upper mixed layer deepened (Figs. 10b, S6). Near‐surface dissipation rates declined as the winds dropped. The up to 0.4 m overturns below the surface and within the upper mixed layer were likely the result of turbulence from shear driven by horizontal advection.

The coefficient of eddy diffusivity, *K*_*z*_, exceeded 10^−5^ m^2^ s^−1^ in the mixed layer and on windier days was up to 10^−3^ m^2^ s^−1^ (Figs. 5, 6, 7, 8, 9, 10). Below the mixed layer, *K*_*z*_ declined to values of molecular diffusivity, 10^−7^ m^2^ s^−1^. *K*_*z*_ was of order 10^−5^ m^2^ s^−1^ at the base of the epilimnion on days with lighter winds and an order of magnitude higher on windier days when temperature inversions indicative of Kelvin–Helmholtz billows were present. During nocturnal cooling, only the calculated values below 0.5 m are meaningful. Values computed as K_z_ = *b N* ^−2^ (*see* Methods) during times without surface overflows are the same order of magnitude as those in Fig. 7.

### Near‐surface turbulence

**Fig. 11:**
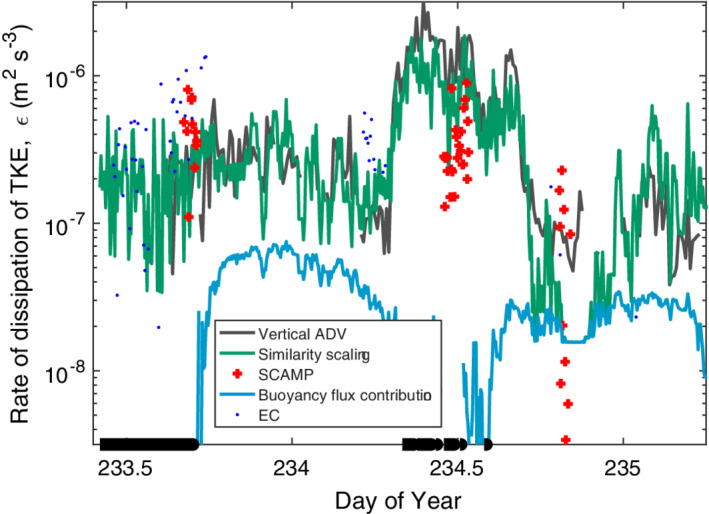
Time series of rate of dissipation of turbulent kinetic energy as measured by the ADV (black), the SCAMP (red +), and computed from the similarity scaling in Tedford et al. (2014) using wind and buoyancy flux (*β*) (green) and only *β* (cyan). Computed *ε* from the eddy covariance wind speed data when it met quality controls as in Fig. S3 (blue dots). Calculated *ε* are at depth of measurement volume of the ADV (*z*_ADV_). Doys 233 to 235 with *z*_ADV_ at 0.15 m. Here and in the following, data from the SCAMP are from the uppermost bin which is approximately the upper 0.25 m of the water column.

**Fig. 12:**
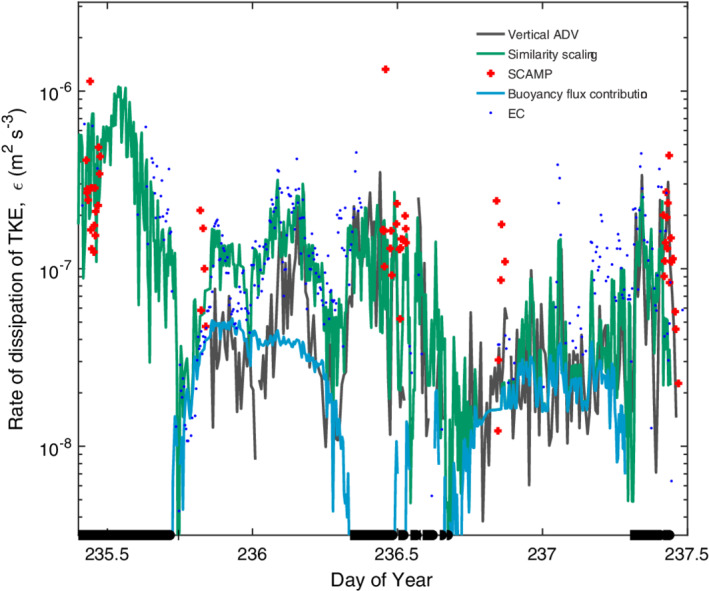
As in Fig. 11 but for doys 235–237 and *z*_ADV_ at 0.25 m.

Dissipation rates obtained with the ADV varied with changes in wind speed over the course of the day (Figs. 11, 12). Dissipation rates were higher, 10^−6^ m^2^ s^−3^, when winds were elevated on the afternoon of doy 234 and values were lower, 2 × 10^−7^ to 3 x 10^−7^ m^2^ s^−3^, on the two nights when winds were 1–2 m s^−1^. With the slightly deeper measurement volume on the second deployment, values of *ε* were lower but still followed the pattern of higher values in the day when winds were elevated and lower values at night when winds had decreased. Dissipation rates obtained with temperature‐gradient microstructure profiling were similar in magnitude to those obtained from the ADV but more variable during each sampling period. During the windy periods, the difference may result from sampling at different locations or advection of water with slightly different mixing regimes. At night, the order of magnitude variability resulted from near‐surface turbulence being generated by convection and by advection (Figs. 7, 11, 12).

Dissipation rates measured by the ADV tracked those computed from the similarity scaling indicating that the turbulence was primarily generated by meteorological forcing (Figs. 11, 12). Dissipation rates computed using the wind speeds measured at the EC station, *ε*_EC_, were also similar to those from the ADV. Departures occurred, as on doy 233.5, when values of *ε*_EC_ exceeded those from the ADV but were similar to values obtained with the microstructure profiler. These differences point to spatial variability in the wind field over the lake. Measured dissipation rates and *ε*_*z*_ from the similarity scaling exceeded *ε*_*β*_ most of the time indicating the turbulence was primarily caused by wind shear not by heat loss. Late on doy 235 and on the night of doys 236 to 237, *ε* and *ε*_*β*_ were equivalent indicating that near surface turbulence was caused by convection due to heat loss at those times. During the late morning sampling on doy 237, dissipation rates from the ADV and the microstructure profiler were intermittently an order of magnitude higher than the values predicted by the similarity scaling using both the weather station data and the EC data. This time period was the only one in which *z*/*L*_MO_ was consistently above 0.1 when we sampled (Fig. S8).

### Relation of *ε* to wind speed

**Fig. 13:**
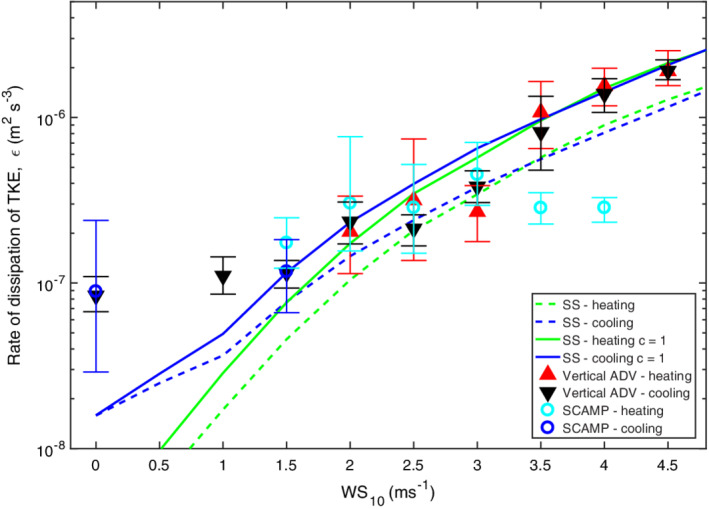
Wind speed at 10 m height calculated taking into account atmospheric stability (WS_10_) vs. maximum likelihood estimate (MLE) of dissipation rate (*ε*) from the ADV (heating, red triangles; cooling, black triangles), the SCAMP (cooling, blue o; heating, cyan o), calculated from the similarity scaling, SS, following Tedford et al. (2014) for cooling (blue dashed) and heating (green dashed), and following Tedford et al. (2014) with a coefficient of 1 for the shear term under cooling (blue) and under heating (green). Only ADV data from the first deployment were used, and calculations for the similarity scaling are for 0.15 m depth. MLE and 95% confidence limits were only computed when the number of data points per bin exceeded 3.

Dissipation rates computed from the ADV under both heating and cooling were similar to those predicted from the similarity scaling for the range of wind speeds in this study (Fig. 13). Predictions were slightly improved using coefficients of 1 for the shear term rather than the 0.56 and 0.6 in Tedford et al. (2014). The difference, however, is less than a factor of two and will have minor influence on the calculation of gas transfer velocities. Under cooling for winds less than 1 m s^−1^, binned dissipation rates computed from the ADV and the SCAMP averaged 10^−7^ m^2^ s^−3^, higher than the 10^−8^ m^2^ s^−3^ predicted from the similarity scaling or from buoyancy flux (*ε*_*β*_, 2 × 10^−8^ to 6 × 10^−8^ m^2^ s^−3^). The discrepancy results from uncertainty in the magnitude of the wind when it is below instrument threshold and to shear from residual surface currents (Figs. 7, 11). During heating, mean *ε* from the SCAMP for winds of 1.5 and 2.0 m s^−1^ were elevated relative to *ε*_*z*_. Mean winds of that magnitude occurred on doys 233, 236, and 237 during heating. The discrepancy may result from higher winds over the lake than over the wetland (e.g., Figs. 11, 12, S3). However, on doy 237, dissipation rates from both the SCAMP and the ADV occasionally exceeded predicted values from the similarity scaling when *L*_MO_/*z*_AML_ < 1 and *z*/*L*_MO_ was above 0.1 (Figs. 10, 12, S8). At such times, buoyancy flux contributes to turbulence by increasing near‐surface shear (Wyngaard and Coté 1971; Tedford et al. 2014, eq. 1, table 2).

### Gas transfer velocities

**Fig. 14:**
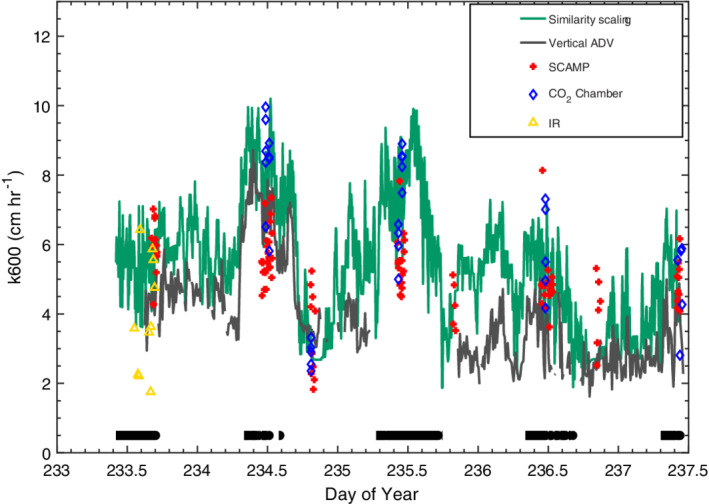
Time series of *k*_600_ computed using the surface renewal model and dissipation rates computed from the ADV (*z*_ADV_ = 0.15 m initial deployment, 0.25 second deployment), the SCAMP, the similarity scaling at 0.15‐m depth with the coefficients for the shear term set equal to 1 (*see* Fig. 13), computed from the chamber measurements, and from surface divergence measurements with the IR camera. Black underbars indicate periods with heating.

Values of *k*_600_ computed with the surface renewal model using *ε* from the similarity scaling and the ADV are similar during both day and night as would be expected from the comparisons of the time series of dissipation rates (Figs. 11, 12, 13, 14). Similar values were also computed from the IR system on doy 233. Gas transfer velocities were highest during afternoon winds, with maximal values of 10 cm h^−1^. Values at night were 5 cm h^−1^ when winds were around 2 m s^−1^ and 3 cm h^−1^ when winds were ~ 1 m s^−1^. For the nights of 235–236 and 236–237, dissipation rates were similar to those predicted from buoyancy flux, and values averaged ~ 3 cm h^−1^ (Figs. 12, 14).

The *k*_600_ obtained from dissipation rates from the SCAMP and from inverse procedures with the chamber measurements closely agree and have similar variability over each sampling period. Similar to the results from the time series data, values were elevated during afternoon winds and lower at night. Variability was up to twofold during each measurement interval but similar to the observed range of *k*_600_ over slightly longer intervals from the time series calculations.

Gas transfer velocities obtained with the IR system when it was upwind, and therefore the water surface was sheltered, were approximately two times less than at the more exposed sites. Average values of *k*_600_ were 3.3 cm h^−1^ mid‐day on doy 235, 1.7 cm h^−1^ on the evening of doy 235, and 2.5 cm h^−1^ on mid‐day 237.

### CO_2_ concentrations and fluxes

Concentrations of CO_2_ in surface water varied with some of the change likely dependent on meteorological conditions and resulting within lake mixing (Table 1, Figs. 2, 3, 4, 5, 6, 7, 8, 9, 10). From noon on doy 234 until early evening, CO_2_ concentrations increased from 70 to 98 μM coinciding with the cloudy, windy conditions with low *L*_*N*_, which led to mixed layer deepening and the increased temperature at 1 m (Figs. 2, 3, 4, 5, 6). Concentrations measured with CO_2_ chambers continuously deployed on the lake had diel variability of 15 μM later in the study. The larger change on doy 234 may have resulted from vertical transport, though we do not have profile data to validate this inference. Fluxes were among the highest measured during the study on doy 234, pointing to the enhanced turbulence both increasing near‐surface concentrations and gas transfer velocities (Figs. 3g, 6, 14). The CO_2_ fluxes estimated by the chambers ranged from 2.1 to 5.1 mmol m^−2^ h^−1^. As *k*_600_ measured by the chambers varied threefold and surface concentrations only 30%, most of the variability in fluxes was explained by *k*. The lowest chamber *k*_600_ estimates and lowest fluxes occurred early evening of day 234 when, with negligible winds, near‐surface turbulence was due to convection and residual currents (Figs. 2c–f, 7). Highest emissions occurred during the second sampling period doy 235 which was similarly windy to doy 234 albeit stratified (Figs. 3f, 8). Fluxes were slightly lower the following 2 days with lighter wind, lower values of *k*, and more variable near‐surface dissipation rates as the near‐surface alternately heated and cooled (Figs. 9, 10, 14). Fluxes measured with the EC system on doys 235.43–235.57, the only interval when quality controls for CO_2_ emissions were met, averaged 4.7 ± 3.6 mmol m^−2^ h^−1^, similar to fluxes estimated by chambers and the surface renewal model (Table 1). Measured fluxes and those modeled with the surface renewal model were similar whereas those estimated using the wind‐based model of Cole and Caraco (1998) were half as large (Table 1).

**Table 1:** CO_2_ concentration (μM), fluxes (*F*, mmol m^−2^ h^−1^), standard deviation of *F* (mmol m^−2^ h^−1^), *k*_600_ (cm h^−1^) derived from chamber data and standard deviation of *k*_600_ (cm h^−1^), and flux using wind‐based model of Cole and Caraco (1998) assuming the atmosphere was neutral (flux CC neut) and correcting for atmospheric stability (flux CC unst), and flux with the surface renewal model with dissipation computed following Tedford et al. (2014) (flux SR). The final column indicates ambient conditions and, when windy, hourly averaged Lake number (*L*_*N*_), and Monin–Obukhov length scale (water side) divided by mixing layer depth (*L*_MO_/*z*_AML_). Means and medians are for the seven data sets.

Day of year	CO_2_	*F*	SD	*k*_600_	SD	Flux CC neut	Flux CC unst	Flux SR	Conditions
234.49	70	4.3	0.7	8.6	1.4	1.5	2.0	4.2	Windy, *L*_*N*_ ~ 3, |*L*_MO_/*z*_AML|_ > 2
234.51	70	3.92	0.7	7.9	1.4	1.7	2.5	5.0	Windy, *L*_*N*_ ~ 3, |*L*_MO_/*z*_AML_| > 2
234.81	98	2.1	0.3	2.8	0.4	2.1	2.1	2.3	Cooling, *β* < 10^−7^ m^2^ s^−3^
235.43	92	3.9	0.4	6.0	0.7	2.6	2.9	5.8	Windy, *L*_*N*_ ~ 4, *L*_MO_/*z*_AML_ > 2 but T‐data show sign change
35.46	88	5.1	0.3	8.3	0.5	1.3	2.5	4.9	Windy, *L*_*N*_ ~ 4, *L*_MO_/*z*_AML_ > 2
236.48	82	3.2	0.7	5.8	1.3	1.7	1.9	3.6	Less windy, *L*_*N*_ ~ 10 *L*_MO_/*z*_AML_ variable
237.42	83	2.8	0.8	5.0	1.5	1.5	1.5	2.6	Less windy, *L*_*N*_ ~ 10
Mean	83	3.6	0.6	6.4	2.1	2.0	2.1	4.1	
Median	83	3.9		6.0		1.9	2.0	4.2	

## *Discussion*

Our study shows that small, sheltered lakes can be dynamic systems with near‐surface turbulence similar to that in larger water bodies and following the same scaling laws. We obtained similar values of near‐surface turbulence, gas transfer velocities, and fluxes of CO_2_ using a diverse suite of measurement and modeling techniques. These approaches provide validation that near‐surface turbulence can be computed in small lakes using MOST; that gas transfer velocities depend on turbulence and that even in small lakes they are primarily dependent on wind shear during cooling; the timing of mixing events scales as predicted from dimensionless indices; and wind‐induced upwelling and downwelling of diurnal and seasonal thermoclines indicate pronounced three dimensionality of movements in small lakes. We also provide the first demonstration that an IR camera can quantify surface divergence from which gas transfer velocities can be computed in small water bodies. In the following, we describe the implications of these measurements of turbulence, meteorology, and time series temperatures for calculating gas transfer velocities and vertical and horizontal fluxes within small lakes. We compare our models of gas transfer velocities with others to further identify critical drivers.

### Near‐surface turbulence

As expected for a small, sheltered lake with high concentrations of DOC (22 mg L^−1^) and *k*_d_ = 2 m^−1^, the upper mixed layer of Lake Övre Björntjärn was shallow in summer. Measured *ε* scaled with predictions from similarity scaling developed for larger water bodies. Values reached 10^−6^ m^2^ s^−3^ in surface waters when winds were only 4 m s^−1^ and were persistently above 10^−7^ m^2^ s^−3^ when winds were only 2 to 3 m s^−1^ (Figs. 11, 12, 13). The high values are the same order of magnitude as in similarly sized North Lake, Australia, in a 22.2‐km^2^ sub‐basin of Lake Opeongo, Canada, and Lake Pleasant, United States, under similar wind speeds (MacIntyre 1993; Pernica et al. 2014; Tedford et al. 2014) but an order of magnitude less than in 150 km^2^ Mono Lake when winds reached 10 m s^−1^ (MacIntyre et al. 1999). During daylight hours on the 2 days with moderate winds, that is, when wind speeds were sustained above 3 m s^−1^, the Monin–Obukhov length scale divided by mixed layer depth (*L*_MO_/*z*_AML_) indicated that wind forcing dominated over buoyancy forcing during both heating and cooling in the actively mixing layer. *ε* exceeded 5 × 10^−8^ m^2^ s^−3^ through much of the mixed layer. On the other 3 days, winds were lighter, diurnal thermoclines formed, and |*L*_MO_/*z*_AML_| was sometimes less than 1. On those days, *z*/*L*_MO_ was at times greater than 0.1 (Fig. S8), where *z* is 0.15, conditions indicating positive buoyancy flux would have augmented turbulence production in the upper mixing layer.

Turbulence measured with the ADV scaled with that computed using the similarity scaling with improvement using a coefficient of 1 for the shear term (Fig. 13). Measured dissipation rates were similar to those measured using similar approaches in a small pond (MacIntyre et al. 2018). The slightly higher dissipation rates relative to the similarity scaling for winds of 1 m s^−1^ or less during cooling were either due to higher winds over the lake relative to the wetland (Figs. S1, S3), uncertainty in wind speed when measured values were less than the anemometer's threshold, or shear from residual currents after the wind ceased (Fig. 2e). Dissipation rates with the microstructure profiler during heating with winds averaging 1.5–2 m s^−1^ are slightly elevated above the similarity scaling following Tedford et al. (2014), but such could occur if *z*/*L*_MO_ > 0.1 (Wyngaard and Coté 1971; Grachev et al. 2013) and imply increased near‐surface shear under light winds and heating. The correspondence between measured and modeled dissipation rates indicates little sheltering by trees in contrast with evaluations elsewhere (Markfort et al. 2010) and that the clearing around the weather station enabled it to measure winds similar to those on the lake.

Dissipation rates measured with the SCAMP were more variable near the surface over hourly intervals than those obtained with the ADV (Figs. 11, 12). Near‐surface temperature varied between casts indicating the variability in *ε* was due, in part, to advection of different water masses and our sampling in different locations (Figs. 5, 6, 7, 8, 9, 10).

### Gas transfer velocities

Our study is the first to compare gas transfer velocities obtained with chamber measurements and the surface divergence model with ones computed from the surface renewal model computed using ε from ADV and microstructure data and calculated from similarity scaling. As such, our results support the growing literature showing the value of turbulence‐based equations for estimation of *k* (Zappa et al. 2007; Wang et al. 2015) and provide a path to derive time‐series calculations of *k* from meteorological measurements.

**Fig. 15:**
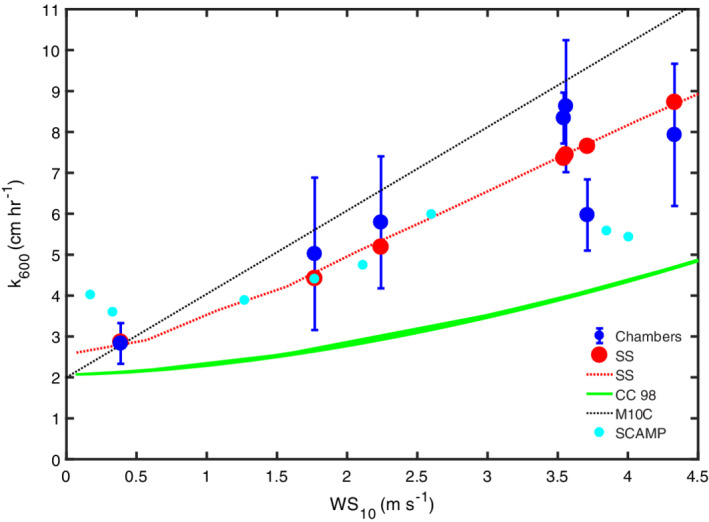
*k*_600_ vs. wind speed at 10 m height computed taking into account atmospheric stability (WS_10_) and averaged for 30 min from the start of the chamber deployment. *k*_600_ from (1) chamber deployments using inverse methods (blue) with bars showing 95% confidence intervals, (2) SS: Surface renewal model with dissipation rate from similarity scaling with coefficient of 1 for the shear term (red dots at time of chamber measurements and red dotted line for the range of wind speed over the experiment), (3) CC98: Wind based model of Cole and Caraco (1998) (green, range of wind speeds); (4) regression model of MacIntyre et al. (2010) under cooling: *k*_600M10c_ = 2.04WS_10_ + 2 (black dotted line); (5) surface renewal model using dissipation rates measured by SCAMP (cyan dots; winds were averaged over period of profiling). Regression equations for the similarity scaling using the range of wind speeds over the experiment: *k*_600SS_r_ = 1.49WS_10_ + 2.1; using winds averaged over each chamber deployment: *k*_600SS_c_ = 1.52WS_10_ + 2.0; and regression equation based on the chamber measurements excluding the outlier from the first sampling on doy 235: *k*_600ch_ = 1.45WS_10_ + 2.6. Units are cm h^‐1^.

Gas transfer velocities increased linearly as a function of wind speed, and the agreement was excellent between the regressions of *k*_600_ from chamber data and from the similarity scaling (Fig. 15). The dependence averages to *k*_600_ = 1.5 *U*_10_ + 2.0, where *U*_10_ is wind speed at 10 m corrected for atmospheric stability.

**Fig. 16:**
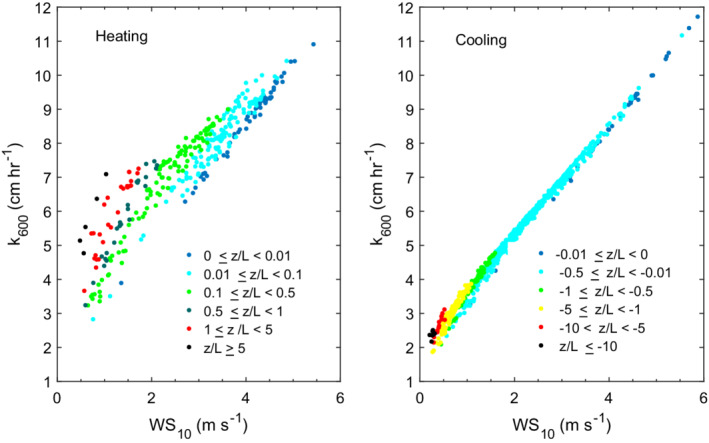
Wind speed at 10 m height (WS_10_) vs. *k*_600_ computed using the surface renewal model with *ε* calculated using the similarity scaling *ε* = *ε*_s_ (1 + 2.5 |*z*/*L*|^3/5^)^1.5^ under heating and *ε* = *ε*_s_ (1 + 0.5 |*z*/*L*|^2/3^)^1.5^ under cooling (Wyngaard and Coté 1971). *L* = *L*_MO_ calculated from the surface energy budget for this study using wind speed from the eddy covariance station. For the smallest ratio of *z*/*L*_MO_, *ε* = *ε*_s_ = *c* $u_{*w}^{3}$ / 0.4*z* and follows law of the wall scaling. Here *c* = 1. The influence of buoyancy flux on *ε* increases as *z*/*L*_MO_ increases. We obtained similar results during heating following Grachev et al. (2013) assuming Richardson numbers were critical for turbulence.

Gas transfer velocities can be higher than predicted from wind‐based relations when winds are light under heating conditions (Fig. 16a). This result follows when the equations for similarity scaling explicitly include the dependence on *z*/*L*_MO_ (Grachev et al. 2013; Tedford et al. 2014). With light winds, the downward movement of momentum is suppressed during heating, and the near‐surface shear produced by a given wind stress increases (Thorpe 2007). For |*z*/*L*_MO_| < 0.01, predicted values of *k*_600_ increase linearly with wind speed (Fig. 16). However, under heating, as the ratio of *z*/*L*_MO_ becomes progressively larger, values of *k*_600_ increase (Fig. 16a). For example, for winds of 1.5 m s^−1^, as *z*/*L*_MO_ increases from 0.01 to 5, *k*_600_ increases from 4 to 7 cm h^−1^. Under cooling, the increase in *ε* was smaller as *z*/*L*_MO_ increased (Fig. 16b). The range of *k*_600_ obtained due to variations in |*z*/*L*_MO_| is similar to the range expected from the standard deviations of the chamber measurements. The enhancement of *k*_600_ relative to wind‐based predictions in Heiskanen et al. (2014) during seasonal stratification is similar to predictions in Fig. 16. McGillis et al. (2004) also found CO_2_ flux enhanced over predictions from wind based models when diurnal thermoclines developed. Additional effort is required to verify coefficients during heating in stratified water bodies.

Our results differ from those indicating convection would dominate turbulence production in lakes smaller than about 10 ha (Read et al. 2012). We found that shear contributed to turbulence production even under cooling, and *k*_600_ exceeded expectations from buoyancy flux due to heat loss except on nights when winds decreased below 0.8 m s^−1^. The analysis based on *z*/*L*_MO_ indicates the enhancement in *ε* as heat loss increases is minor although some variability is expected based on the multiplier on *z*/*L*_MO_ (Fig. 16b).

Once wind speeds exceeded 1 m s^−1^, *k*_600_ was 2–3 times higher than estimates computed following Cole and Caraco (1998) (Fig. 15). Lower values obtained following Cole and Caraco (1998) likely result from their tracer studies including days with stable stratification in the atmosphere over the lakes and more frequent periods with stronger near‐surface stratification than in this study. Predicted values are less than in the equation derived for cooling in MacIntyre et al. (2010) (Fig. 15). Gas transfer velocities were higher than predicted for a 1 ha lake and similar to predictions for 1 km^2^ lakes in models based on wind speed and lake area (Vachon and Prairie 2013; Klaus and Vachon 2020). This difference implies that other factors than significant wave height are key drivers for small lakes. As discussed above, these include atmospheric stability, residual currents under cooling, and the contribution of buoyancy flux particularly under heating. While our chamber and ADV measurements were mid‐lake in Övre Björntjärn, the similarity in fluxes with the eddy‐covariance system and chambers indicates that our results apply over a larger surface area than would be expected based on the siting of the chamber measurements.

Unsteady winds and currents in the lake may modify gas transfer velocities expected from the linearity with respect to wind (Fig. 15). The differing conditions during our first and second SCAMP and chamber deployments on doy 235 provide an example. Fluxes computed with the chambers were lower than expected during the first sampling period when wind speed was unsteady and wind direction shifted from south to north. SCAMP derived estimates of *k*_600_ were also low at that time (Table 1, Fig. 14). During the second deployment, wind speed and direction were more stable, and *k*_600_ from the chambers followed the linearity illustrated in Fig. 15.

The *k*_600_ obtained from dissipation rates from the SCAMP and from inverse procedures with the chamber measurements closely agreed and had similar variability over the sampling period (Fig. 14). Variability was up to twofold over short times and distances. Jointly these results indicate that variability in *k* is real and common. In addition to unsteady winds, variability can be caused by residual currents, the heterogeneity between water patches in energy input (e.g., wind exposure which is often visually patchy across lakes), additional processes causing flows such as differential heating and cooling, and as evaluated in Fig. 16, the relative influence of wind and buoyancy flux. Variability can also result from advection causing differences in surface concentrations over small spatial scales. These results point to the necessity of replicated chamber measurements when estimating emissions, as is also required when addressing temporal variability (Jansen et al. 2020). Inclusion of the underlying hydrodynamics when estimating fluxes using Eq. 1, *F* = *k* (*C*_w_ *– C*_eq_), provides an approach to constrain the variability.

### Coefficient for the surface renewal model

When *k*_600_ is computed using the surface renewal model, there is uncertainty in the coefficient used as a multiplier (*see* MacIntyre et al. 2019, Appendix S1). Here we have used a value of 0.5. The agreement between *k*_600_ from the chambers and surface divergence method in comparison to values obtained by calculating *k*_600_ with the surface renewal model using dissipation rates from the ADV, the SCAMP, and the similarity scaling supports the use of 0.5 when winds are light to moderate and measurement depth is 0.15 m (Figs. 14, 15).

### Quantifying surface divergence with an IR camera

Estimates of *k*_600_ with the surface divergence model agreed with those from the surface renewal model and using inverse methods with chambers when winds were 4 m s^−1^ (Fig. 14). Values obtained when the measurement site was in the ~ 10 m long wind shadow are similar to those obtained from residual currents when winds decreased. This good agreement indicates that an IR camera, such as used here, can provide estimates of *k*_600_ when winds are moderate and wave heights low. These results contrast with those in Gålfalk et al. (2013) when significant wave heights were between 0.1 and 0.3 m for much of the study such that velocities associated with upwelling features were smeared by the orbital velocities from surface waves. In addition, in this study the camera was deployed closer to the lake surface improving resolution. Typically, success with the surface divergence model has required use of particle image velocimetry with its higher resolution (Turney and Banerjee 2013; Wang et al. 2015; Turney 2016). Thus, the results here are the first successful demonstration that velocities of upwelling features can be captured with an IR camera enabling calculation of *k* using the surface divergence model.

### Mixing within the epilimnion

The upper mixed layer was always turbulent during our study (Figs. 5, 6, 7, 8, 9, 10). *K*_*z*_ often exceeded 10^−4^ m^2^ s^−1^ near the surface and decreased by approximately a factor of 5 below. On the days with the weakest winds, *K*_*z*_ at the surface was ~ 5 × 10^−5^ m^2^ s^−1^. On windy days, *K*_*z*_ sometimes increased at the base of the mixed layer indicating increased shear. Variability in dissipation rates and *K*_*z*_ with depth and time in the mixed layer at night was due to intermittent surface overflows which were mixing independently of the water below. Immediately below the upper mixed layer, *K*_*z*_ ~ =10^−7^ m^2^ s^−3^, close to molecular conductivity.

To influence calculations of metabolism, vertical fluxes must occur on the time scale of biological changes. The time scale of mixing *τ*_mix_ equals *l* ^2^/*K*_*z*_, where *l* can be the depth of the surface layer or the distance between the top of the thermocline and the base of the mixed layer. For a 1‐m thick mixed layer and *K*_*z*_ = 10^−5^ m^2^ s^−3^, *τ*_mix_ = 1 d. For *K*_*z*_ = 10^−4^ m^2^ s^−3^, *τ*_mix_ = 3 h. The higher values of *K*_*z*_ occurred when *L*_MO_/*z*_AML_ was more consistently above 1. Values decayed as *L*_MO_/*z*_AML_ approached 0 and the turbulence worked against a stratified water column. Under penetrative convection, the mixing time scale can be calculated as *l*/w_*_, which for a 1‐m deep surface layer and the typical w_*_ at night of 0.004 m s^−1^ (Fig. 2e), would be 4 min. Using *τ*_mix_ = *l* ^2^/*K*_*z*_ and *K*_*z*_ = 10^−3^ m^2^ s^−1^, the time scale is only somewhat longer. The time scales within the mixed layer are rapid enough that CO_2_ or CH_4_ entrained from deeper waters or transported across the lake with afternoon winds will quickly reach the air–water interface and evade.

Fluxes into the mixed layer would occur during upwelling (Figs. 4, S7) when Lake numbers drop to values low enough to create shear between the mixed layer and thermocline (Fig. 3b,g). Larger overturns and elevated *ε* were observed at depths with intrusions of cooler water (Fig. 8c,d). For a 0.5‐m overturn and *K*_*z*_ = 2 × 10^−4^ m^2^ s^−1^, the mixing time scale would be 20 min. Multiple such events occurred on day 235 (Fig. 8e), implying exchanges could occur from the base of the mixed layer upward. Fluxes are also expected with entrainment during cooling events (Czikowsky et al. 2018). However, computed deepening following Turner (1973, see methods) from the 14 hour period from late afternoon on doy 234 through the morning of doy 235, when *β* averaged 4 × 10^−8^ m^2^ s^−3^, was less than 1 cm. Calculations following Monismith (1986) indicate shear induced entrainment would have been minimal for the same period. For strongly stratified lakes, these calculations point toward the importance of upwelling‐induced horizontal advection and subsequent mixing of the intruding water for vertical exchange.

*K*_*z*_ can be calculated in the actively mixing layer as a function of depth when ε is computed from similarity scaling (MacIntyre et al. 2018). During microstructure profiling in this study, the ratio of *L*_MO_/*z*_AML_ tended to exceed 1, and mixing extended below the actively mixing layer. When such occurred, Lake numbers had often decreased to values indicating tilting of the thermocline which produces shear to cause additional mixing. As can be seen on doy 235, temperature inversions at the base of the mixed layer indicated Kelvin–Helmholtz billows were present, and dissipation rates were elevated (Fig. 8b,d). These observations provide a basis for computing *K*_*z*_, again using the algorithms as in Shih et al. (2005) and Bouffard and Boegman (2013), and thus extend modeling of *K*_*z*_ below the actively mixing layer. At night when wind speeds are low, *K*_*z*_ can be computed from the heat budget method (Jassby and Powell 1975; MacIntyre et al. 2018) as long as advection is minor and the time series temperature data are filtered to remove the confounding influence of upwelling and downwelling. Thus, use of the similarity scaling enables calculation of *K*_*z*_ in the dynamic, upper mixing layers of lakes.

### The three dimensionality of small lakes and vertical and horizontal exchanges

Despite the small size of Övre Björntjärn, the base of the mixed layer and upper thermocline upwelled and downwelled even for winds of only 3 m s^−1^ (Figs. 3, 4, and S7). The Lake number dropped into the range for partial upwelling, for example, 2–5, and the frequency of temperature fluctuations in the upper thermocline increased to values indicative of turbulence when *L*_*N*_ dropped to these low values (Fig. 3b,g). The ratio *L*_MO_/*z*_AML_ increased above 1, also indicating wind‐driven mixing extended into the more stratified water below the actively mixing layer. Thus, during windy periods, fluxes between the upper mixed layer and top of the thermocline are enabled in small lakes by coupled processes: upwelling, increased shear at the base of the mixed layer/top of the thermocline, and resulting increased turbulence and higher *K*_*z*_. Concentrations of CO_2_ increased in the mixed layer after several hours with winds sufficient to cause the Lake number to drop below 4 supporting the inference of vertical fluxes (Fig. 3, Table 1). The increase on day 234 also occurred with minimal heating (Fig. 2), pointing to cloudy, windy conditions facilitating mixed layer deepening and fluxes of solutes (Figs. 3, 4) as observed in other stratified boreal and arctic lakes (MacIntyre et al. 2009b; Aberg et al. 2010).

The relative magnitudes of *W* and *L*_*N*_ have been posited to indicate whether upwelling would be confined to diurnal thermoclines in the mixed layer or would also induce full tilting of the thermocline, as with a first vertical‐mode internal wave, or whether second vertical‐mode waves would form (Imberger 1985; Monismith 1986). For Övre Björntjärn, the combination of *W* < 1 and *L*_*N*_ dropping to 2 led to upwelling of the upper thermocline and a second vertical‐mode response across the thermocline. For *W* < 1 and *L*_*N*_ = 10, only the diurnal thermocline upwelled and downwelled. Both metrics indicate cross‐basin exchange in the upper mixed layer. However, as *L*_*N*_ decreases, cross‐basin exchange in the thermocline and upward flux to the upper mixed layer increase. For partial upwelling of the thermocline, as expected for the lower values in this study, the extent to which the upwelled water spreads in the upper mixed layer can be computed as *K*_H_ ~ 3 *h*_2_^.^ u_*w_, where *K*_H_ is a horizontal dispersion coefficient, and *h*_2_ in our case is thickness of the thermocline (Monismith 1986). The time scale for spreading, *τ*_H_ = *L* ^2^/*K*_H_, where *L* is length of the lake. For the stronger winds in this study, *K*_H_ = 0.05 m^2^ s^−1^, similar to values determined empirically for a lake of the size of Övre Björntjärn (Lawrence et al. 1995). The time scale of horizontal spreading would be several days. Thus, while water in the upper mixed layer would flow back and forth with seiching, the upwelled water would not fully spread in the horizontal and concentrations would not become uniform across the lake.

The thermocline in Övre Björntjärn was remarkably responsive to shifts in wind speed and direction. While our linear array captured internal wave movements along only one axis, the abrupt upwelling and downwelling as wind shifted (e.g., doy 233.6, Fig. 3; doy 235.8, Figs. 4, S7) imply that the thermocline may rotate around the lake as winds shift, for instance, from northerly to southerly (Thorpe 1998; Vidal et al. 2013). Near‐surface currents, cross‐basin advection, and shear, initially induced as the wind tilted the thermocline, will be sustained for some period of time afterward from continued seiching. Our ADV data indicated that currents can be sustained for periods of at least an hour and a half, a mechanism that can sustain gas fluxes in the absence of wind. Lastly, while we did not obtain data to quantify these processes, advection from differential heating and cooling would be likely in Övre Björntjärn with its shallow regions to the north. Importantly, the combination of flows will transport water depleted in oxygen or with increased CO_2_ and CH_4_ from sites with higher respiration or storage to other locations within the lake. With elevated afternoon winds and low winds at night, inshore–offshore exchange from the wind‐driven circulation will occur on a daily basis.

Consequently, the three dimensionality of small lakes must be considered when designing experiments. Three dimensionality can be especially important if the near‐shore habitat is more active biologically than offshore waters. The interpretation of eddy covariance studies could be strongly influenced if flows from nearshore at night, when winds have dropped, elevate concentrations of climate forcing trace gases to values in excess of those at a one‐point measurement station (Heiskanen et al. 2014; Podgrajsek et al. 2016).

### Comparisons with other studies

Time series of *k*_600_ values computed from measured and modeled dissipation rates varied from 2 to 10 cm h^−1^ in the small, boreal lake we studied. Measured fluxes were in agreement with those computed using the surface renewal model and, based on the linear dependence of *k*_600_ on wind speed, led to the equation, *k*_600_ = 2.0 + 1.5^.^WS_10_. In the earliest studies demonstrating that lakes contributed to regional carbon budgets, conservative values of 2 cm h^−1^ were used in the modeling (Kling et al. 1991; Richey et al. 2002). The wide range of values for winds ranging from 0 to 4 m s^−1^ illustrates that use of variable and higher values of *k*_600_ is justified even for small water bodies. The mean *k*_600_ for our study is 5.2 cm h^−1^ despite the lake's being small and sheltered by trees. This value is nearly two times higher than the 2.8 cm h^−1^ computed for an unstable atmosphere following Cole and Caraco (1998) and 2.5 times higher than the mean value obtained from 10 lakes ranging from 0.3 to 45 ha in surface area and with mean wind speed 3 m s^−1^ (Cole et al. 2010). Our results support Raymond et al.'s (2013) use of higher values of *k*_600_ for global averaging but also indicate that values when winds are light and near‐surface heating is enhanced by humic substances, phytoplankton, or, in cold regions, by longer ice‐free seasons, may be considerably higher than those currently used in regional averaging. For larger lakes exposed to high winds, it is critical to include the large enhancement in *k* due to breaking surface waves (Brumer et al. 2017). These observations indicate that the contribution from lakes relative to streams in full catchment studies has been underestimated (Lundin et al. 2013; Kokic et al. 2014). Thus, the validation of similarity scaling as in Tedford et al. (2014) in this study, in a small pond (MacIntyre et al. 2018), and using eddy covariance in larger lakes (Heiskanen et al. 2014; Czikowsky et al. 2018) indicates the equations for near‐surface turbulence using MOST will lead to improved assessments of lake metabolism and the contributions of lakes of various sizes to regional and global carbon budgets.

## Conflict of Interest

None declared.

## Supporting information


**Appendix S1.** Supplementary figures.
**Fig. S1**. Comparison of meteorological data from the weather station (blue, 5 min averages) with that from the EC system (orange dots, half hour averages, quality control as in methods) with the EC system at Position 1 through the morning of day 235 and at Position 2 subsequently (Fig. 1). (**a**) Wind speed at instrument height (WS), (**b**), Wind direction (WDir), (**c**) Latent heat flux (LE), (**d**) Sensible heat flux (SE). In panels (**a**) and (**b**), the wind data has only been filtered by wind direction; in panels (**c**) and (**d**), the filtering also included evaluation of spectra.
**Fig. S2**. Comparison of computed effective heat flux using weather data station (blue), and EC data for LE and SE (green). All EC data were interpolated to five minutes and filtering was based on wind direction. Effective heat flux is the sum of net short wave radiation retained in the actively mixing layer and net long wave radiation and latent and sensible heat fluxes.
**Fig. S3**. Comparison of wind speed corrected to 10 m (WS10) using data from the weather station (blue) and using wind from the EC station interpolated to 5‐min intervals when EC data met quality controls with correction for atmospheric stability using air temperature and relative humidity from the calibrated weather station sensors (dots).
**Fig. S4**. Infrared image of the water surface with velocity vectors overlaid (mean current subtracted) at Övre Björntjärn taken from the position marked “IR camera” in Fig. 1b. The field of view is approximately 0.40 m × 0.37 m. The image was taken Aug 20 at 16 : 40 UTC (day 233.69; 233.65 Swedish Standard time) and the wind speed was 1.8 m s^−1^. Dark streaks are extended in the direction of the wind and regions of divergence in rising convection cells are evident as velocity vectors separate (arrow marks one such region).
**Fig. S5**. Time series of isotherms at 0.5°C intervals in the upper mixed layer and upper thermocline (upper panel), u*w (middle panel), and wind direction (WDir) lower panel on day 233. Upwelling and downwelling of the diurnal thermocline occurred with changes in wind. SCAMP profiling was conducted at ~ 233.7 at the transition from heating to cooling. At the start of profiling, *z*/*L*_MO_ had just decreased below 0.1.
**Fig. S6**. Time series of isotherms at 0.1°C intervals at the central station (upper panel), wind speed corrected to 10 m height (blue) and measured wind speed (orange) (middle panel), and wind direction (WDir) with wind data from the EC system. Data illustrate the diurnal mixed layer and its responsiveness to changes in wind speed and direction. SCAMP profiling was conducted ~ 237.415 to 237.47.
**Fig. S7**. Five second averaged temperature contours centered on day 235 to illustrate steep fronted internal waves that result during an event with 45‐min averaged *L*_*N*_ = 2. Upwelling occurs at day 235.61 to the north (upper panel, up arrow) and, on relaxation of the wind to the south (lower panel, up arrow). Thermocline compression occurs to the south with expansion to the north and the converse.
**Fig. S8**. Time series during heating of *z*/*L*_MO_, that is, measurement depth (0.15 m) divided by the Monin–Obukhov length scale on the water side. Computations based on weather station data (blue) and wind speed from EC station and air temperature and relative humidity from the weather station (orange).

**Video S1**. Infrared temperature patterns of a 1‐m^2^ water surface area showing convection cells of rising and sinking water at a wind speed of 0 m s^−1^. This example video was made at the Bornö marine research station in Gullmarsfjorden on the Swedish west coast.

**Video S2** IR temperature patterns of a 1‐m^2^ water surface area showing waves and structure development at a wind speed of 2.5 m s^−1^ in the direction of the extended dark streaks. This example video was made at the Bornö marine research station in Gullmarsfjorden on the Swedish west coast.
