# Supplementary material for: Turbulence in a small boreal lake: Consequences for air–water gas exchange
Source: Limnol Oceanogr. 2020 Nov 24;66(3):827–54. doi: 10.1002/lno.11645 (PMC8048862; doi:10.1002/lno.11645)
Supplement: Supplementary file 1 — Appendix S1. Supplementary figures. Fig. S1. Comparison of meteorological data from the weather station (blue, 5 min averages) with that from the EC system (orange dots, half hour averages, quality control as in methods) with the EC system at Position 1 through the morning of day 235 and at Position 2 subsequently (Fig. 1). (a) Wind speed at instrument height (WS), (b), Wind direction (WDir), (c) Latent heat flux (LE), (d) Sensible heat flux (SE). In panels (a) and (b), the wind data has only been filtered by wind direction; in panels (c) and (d), the filtering also included evaluation of spectra. Fig. S2. Comparison of computed effective heat flux using weather data station (blue), and EC data for LE and SE (green). All EC data were interpolated to five minutes and filtering was based on wind direction. Effective heat flux is the sum of net short wave radiation retained in the actively mixing layer and net long wave radiation and latent and sensible heat fluxes. Fig. S3. Comparison of wind speed corrected to 10 m (WS10) using data from the weather station (blue) and using wind from the EC station interpolated to 5‐min intervals when EC data met quality controls with correction for atmospheric stability using air temperature and relative humidity from the calibrated weather station sensors (dots). Fig. S4. Infrared image of the water surface with velocity vectors overlaid (mean current subtracted) at Övre Björntjärn taken from the position marked “IR camera” in Fig. 1b. The field of view is approximately 0.40 m × 0.37 m. The image was taken Aug 20 at 16 : 40 UTC (day 233.69; 233.65 Swedish Standard time) and the wind speed was 1.8 m s−1. Dark streaks are extended in the direction of the wind and regions of divergence in rising convection cells are evident as velocity vectors separate (arrow marks one such region). Fig. S5. Time series of isotherms at 0.5°C intervals in the upper mixed layer and upper thermocline (upper panel), u*w (middle panel), and wind direction [file LNO-66-827-s002.pdf]

Supplementary Figures.

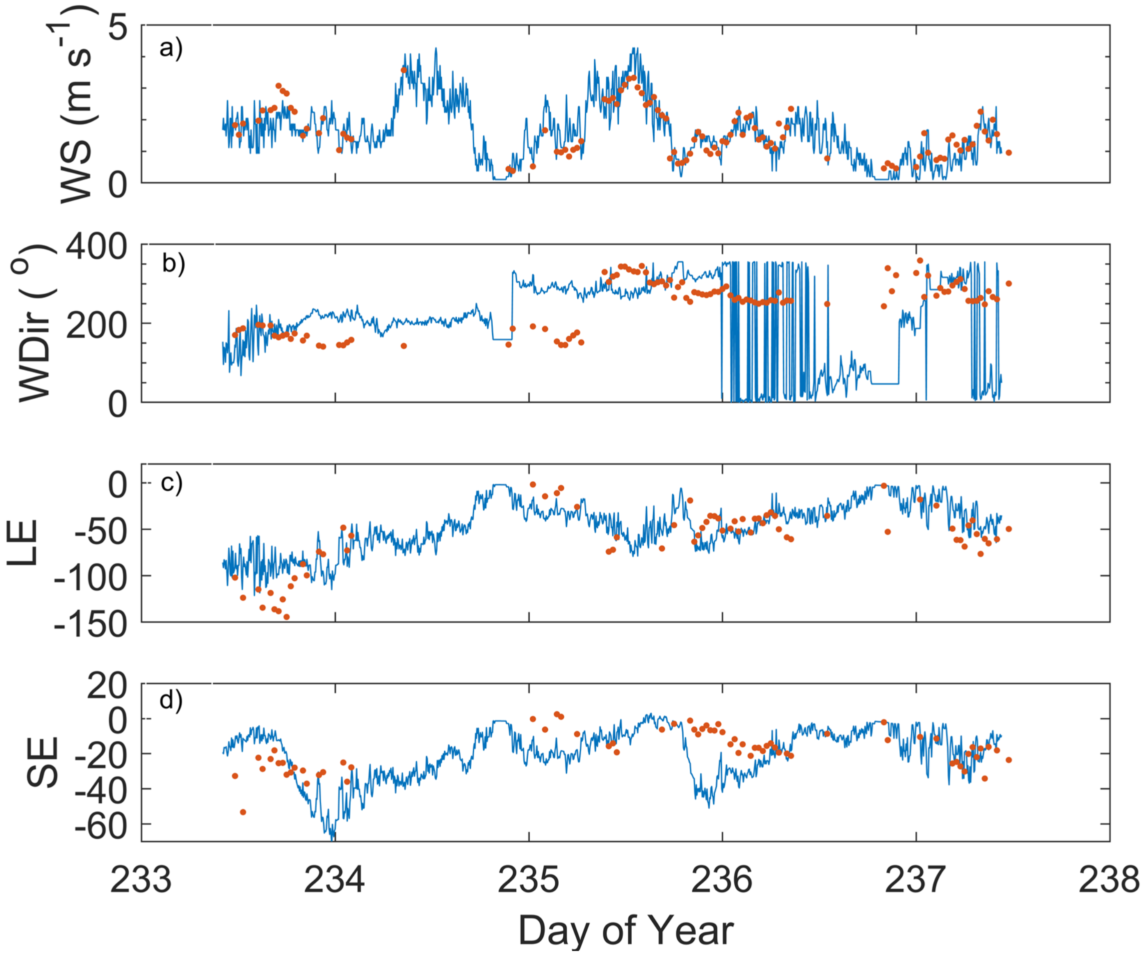

Figure S1. Comparison of meteorological data from the weather station (blue, 5 min. averages) with that from the EC system (orange dots, half hour averages, quality control as in methods) with the EC system at Position 1 through the morning of day 235 and at Position 2 subsequently (Fig. 1). a) Wind speed at instrument height (WS), b), Wind direction (WDir), c) Latent heat flux (LE), d) Sensible heat flux (SE). In panels a and b, the wind data has only been filtered by wind direction; in panels c and d, the filtering also included evaluation of spectra.

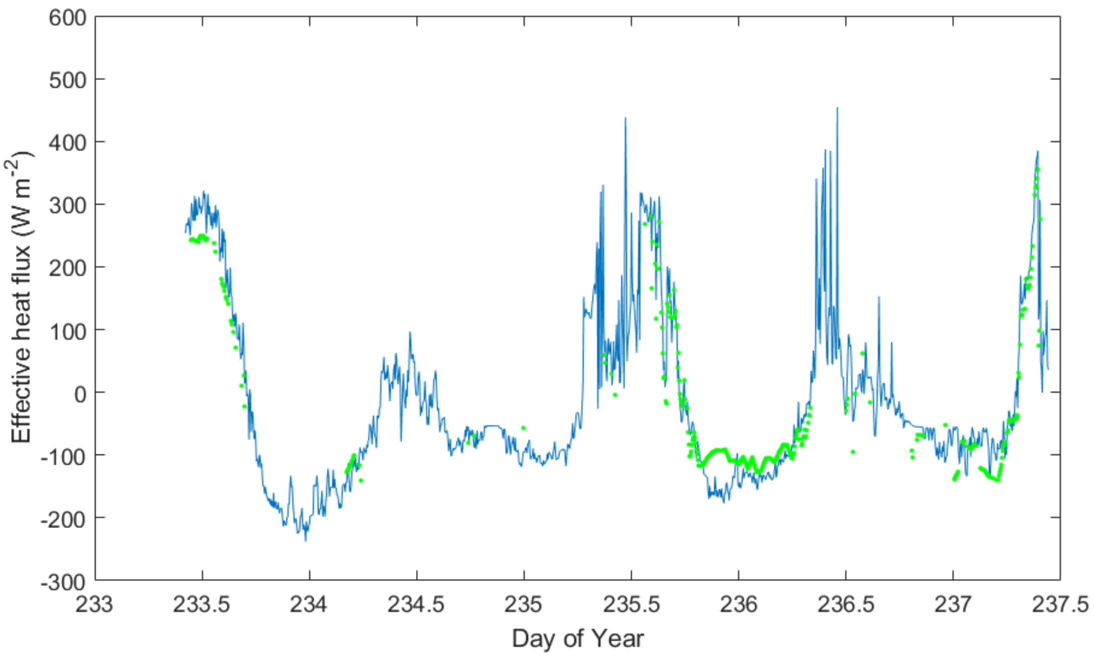

Figure S2. Comparison of computed effective heat flux using weather data station (blue), and EC data for LE and SE (green). All EC data was interpolated to five minutes and filtering was based on wind direction. Effective heat flux is the sum of net short wave radiation retained in the actively mixing layer and net long wave radiation and latent and sensible heat fluxes.

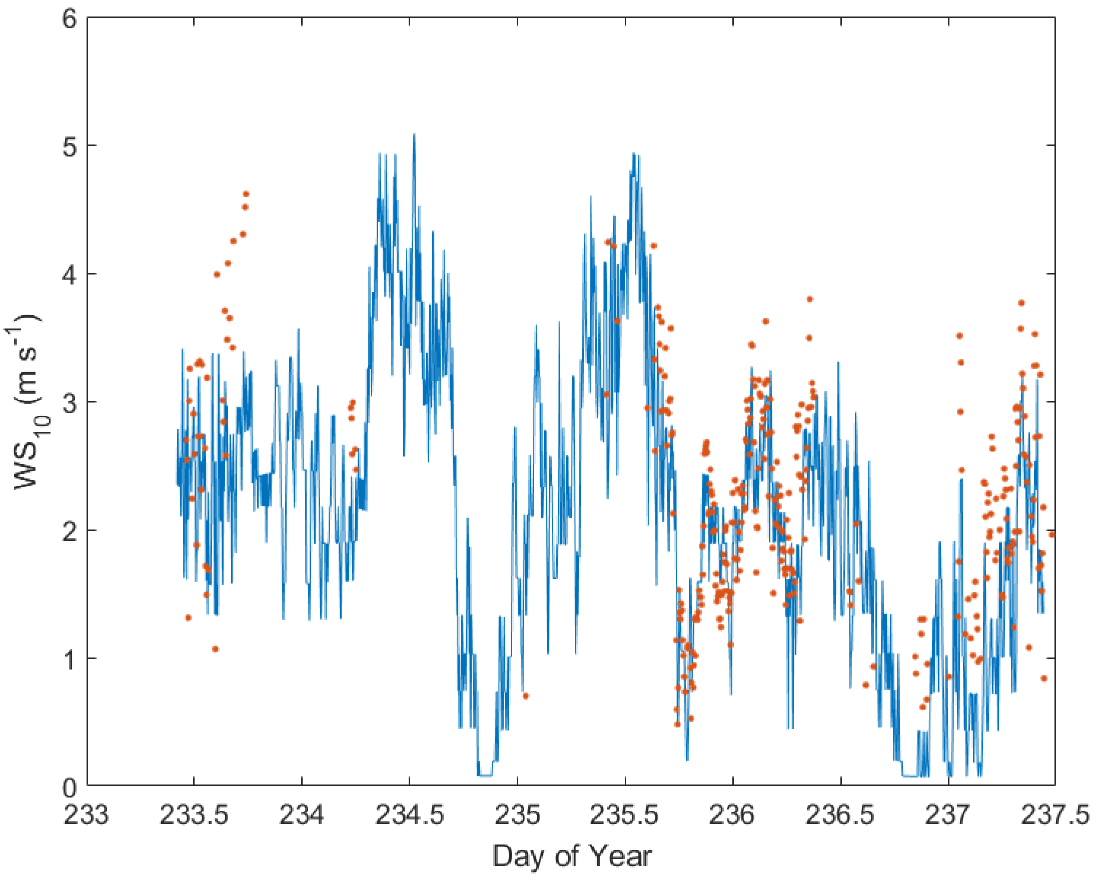

Figure S3. Comparison of wind speed corrected to 10 m (WS_{10}) using data from the weather station (blue) and using wind from the EC station interpolated to 5 minute intervals when EC data met quality controls with correction for atmospheric stability using air temperature and relative humidity from the calibrated weather station sensors (dots).

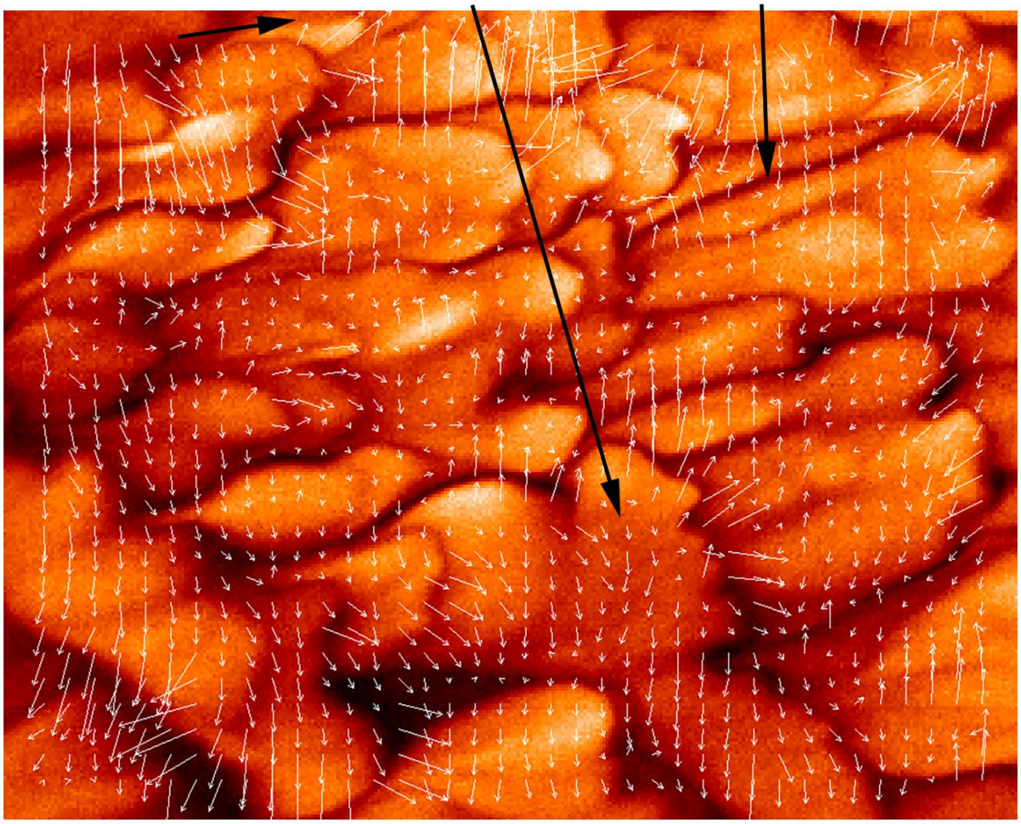

Figure S4. Infrared image of the water surface with velocity vectors overlaid (mean current subtracted) at Övre Björntjärn taken from the position marked 'IR camera' in Fig. 1b. The field of view is approximately 0.40 m x 0.37 m. The image was taken Aug 20 at 16:40 UTC (Day 233.69; 233.65 Swedish Standard time) and the wind speed was 1.8 m s^{-1}. Dark streaks are extended in the direction of the wind and regions of divergence in rising convection cells are evident as velocity vectors separate (arrow marks one such region).

Movies: Examples of IR patterns in different wind conditions.
IR video 1: Infrared temperature patterns of a 1 m^2 water surface area showing convection cells of rising and sinking water at a wind speed of 0 m s^{-1}. This example video was made at the Bornö marine research station in Gullmarsfjorden on the Swedish west coast.
IR video 2: IR temperature patterns of a 1 m^2 water surface area showing waves and structure development at a wind speed of 2.5 m s^{-1} in the direction of the extended dark streaks. This example video was made at the Bornö marine research station in Gullmarsfjorden on the Swedish west coast.

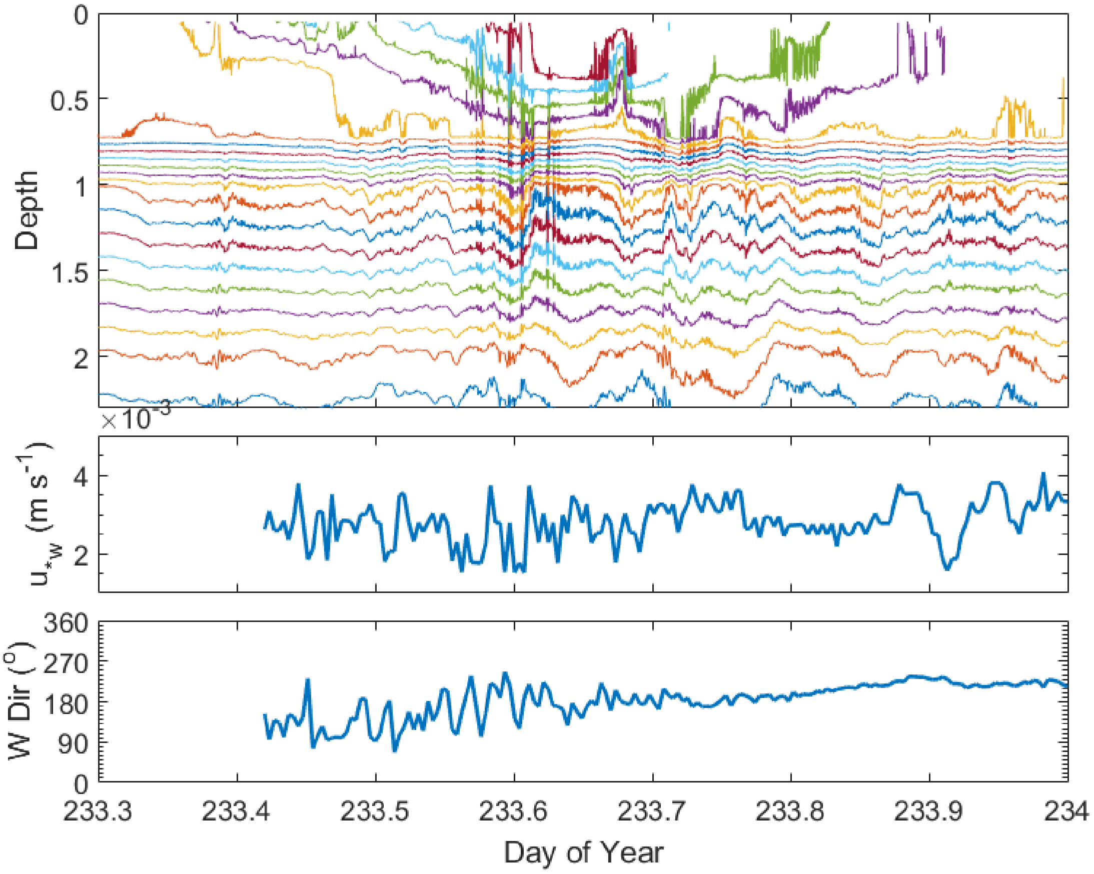

Figure S5. Time series of isotherms at 0.5ºC intervals in the upper mixed layer and upper thermocline (upper panel), u_{*w} (middle panel), and wind direction (WDir) lower panel on day 233. Up and downwelling of the diurnal thermocline occurred with changes in wind. SCAMP profiling was conducted at ~233.7 at the transition from heating to cooling. At the start of profiling, z/L_{MO} had just decreased below 0.1.

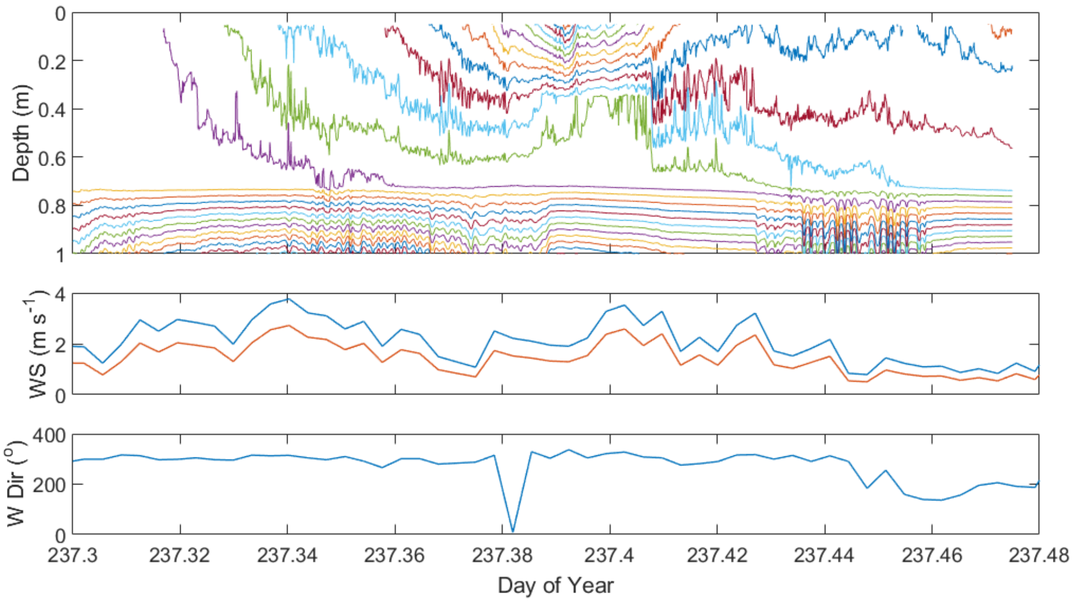

Figure S6. Time series of isotherms at 0.1°C intervals at the central station (upper panel), wind speed corrected to 10 m height (blue) and measured wind speed (orange) (middle panel), and wind direction (W Dir) with wind data from the EC system. Data illustrate the diurnal mixed layer and its responsiveness to changes in wind speed and direction. SCAMP profiling was conducted ~237.415 to 237.47.

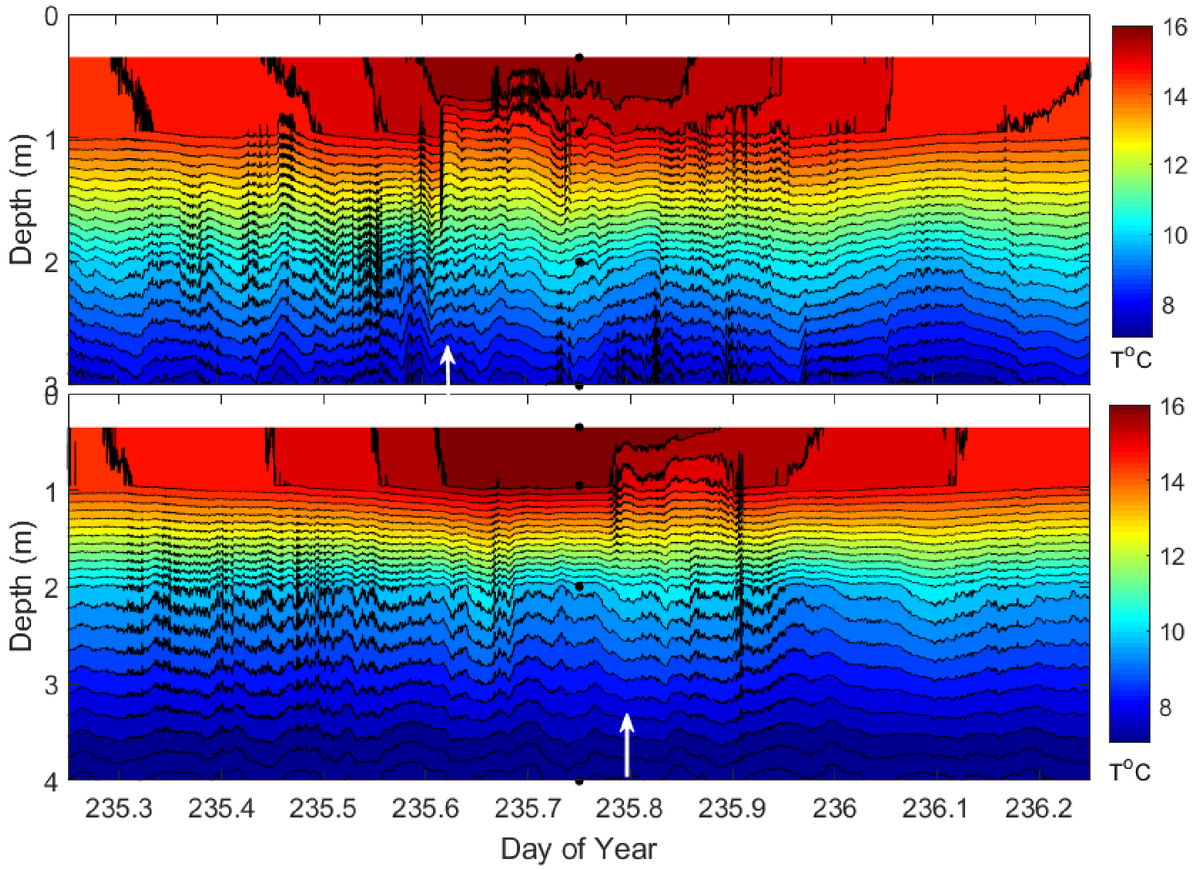

Figure S7. Five second averaged temperature contours centered on day 235 to illustrate steep fronted internal waves that result during an event with 45-minute averaged $L_N = 2$. Upwelling occurs at day 235.61 to the north (upper panel, up arrow) and, on relaxation of the wind to the south (lower panel, up arrow). Thermocline compression occurs to the south with expansion to the north and the converse.

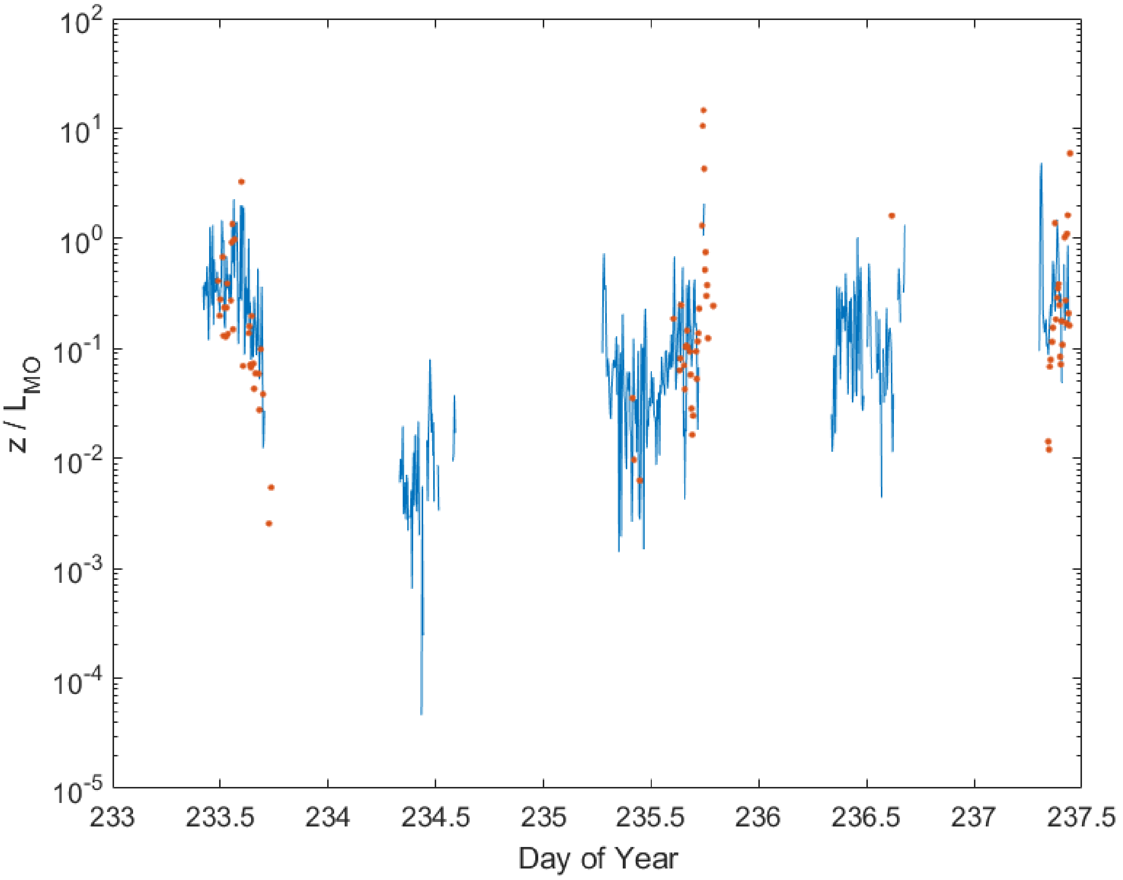

Figure S8. Time series during heating of z/L_{MO}, that is, measurement depth (0.15 m) divided by the Monin-Obukhov length scale on the water side. Computations based on weather station data (blue) and wind speed from EC station and air temperature and relative humidity from the weather station (orange).
